# A taxonomic study of Costa Rican *Leptodrepana* with the description of twenty-four new species (Hymenoptera, Braconidae, Cheloninae)

**DOI:** 10.3897/zookeys.750.23536

**Published:** 2018-04-16

**Authors:** Samin D. Dadelahi, Scott R. Shaw, Helmuth Aguirre, Luis Felipe V. de Almeida

**Affiliations:** 1 U.W. Insect Museum, Department of Ecosystem Science & Management (3354), University of Wyoming, 1000 East University Avenue, Laramie, Wyoming 82071, U.S.A.; 2 Universidade Federal de São Carlos, Departamento de Ecologia e Biologia Evolutiva, Rod. Washington Luis, km 235, CEP 13565-905, São Carlos SP, Brazil

**Keywords:** Carapace, egg-larval parasitoids, Malaise traps, Neotropical Region

## Abstract

The genus *Leptodrepana* Shaw was described in 1983, but prior to the current study only one Neotropical species had been described from Mexico and none were named from Costa Rica. In this paper twenty-four new species are described and named from Costa Rica: *L. alexisae* Dadelahi & Shaw, **sp. n.**, *L. atalanta* Dadelahi & Shaw, **sp. n.**, *L. conda* Dadelahi & Shaw, **sp. n.**, *L. conleyae* Dadelahi & Shaw, **sp. n.**, *L. demeter* Dadelahi & Shaw, **sp. n.**, *L. eckerti* Dadelahi & Shaw, **sp. n**., *L. gauldilox* Dadelahi & Shaw, **sp. n.**, *L. hansoni* Dadelahi & Shaw, **sp. n.**, *L. kimbrellae* Dadelahi & Shaw, **sp. n.**, *L. lorenae* Dadelahi & Shaw, **sp. n.**, *L. munjuanae* Dadelahi & Shaw, **sp. n.**, *L. ninae* Dadelahi & Shaw, **sp. n.**, *L. pamelabbas* Dadelahi & Shaw, **sp. n.**, *L. ronnae* Dadelahi & Shaw, **sp. n.**, L. *rosanadana* Dadelahi & Shaw, **sp. n.**, *L. schuttei* Dadelahi & Shaw, **sp. n.**, *L. scottshawi* Dadelahi, **sp. n.**, *L. shriekae* Dadelahi & Shaw, **sp. n.**, *L. sohailae* Dadelahi & Shaw, **sp. n.**, *L. sorayae* Dadelahi & Shaw, **sp. n.**, *L. soussanae* Dadelahi & Shaw, **sp. n.**, *L. stasia* Dadelahi & Shaw, **sp. n.**, *L. strategeri* Dadelahi & Shaw, **sp. n.**, and *L. thema* Dadelahi & Shaw, **sp. n.** A key to Costa Rican species of *Leptodrepana* is provided. The flagellum of all female *Leptodrepana* described in this work is reduced to only 17 flagellomeres. This character state is also found in two North American species described by Shaw (1983), *L. opuntiae* Shaw and *L. oriens* Shaw. It is hypothesized that a female antenna with 17 flagellomeres is a synapomorphy for a species-group comprising all the Costa Rican *Leptodrepana* species as well as two of the Mexican and North American species, *L. opuntiae* and *L. oriens*.

## Introduction

The braconid subfamily Cheloninae is large and cosmopolitan with more than 1500 described species (Shaw 1997, Yu et al. 2012, Kittel and Austin 2014). Here, the subfamily Adeliinae is considered part of the Cheloninae, in agreement with the results of Kittel et al. (2016). All chelonines are characterized by the presence of a metasomal carapace formed by the fusion of the first three metasomal terga into a rigid structure, dorsally covering the abdomen (Dudarenko 1974). Carapace formation is not unique to the Cheloninae and can be found in many other braconid subfamilies e.g., Microgastrinae, Sigalphinae, Ichneutinae, Rogadinae, and Helconinae (Dudarenko 1974, Shaw 1997). The inclusion of Adeliinae left no obvious morphological synapomorphies to define the Cheloninae. However, it is hypothesized that egg-larval parasitism is a trait unifying the subfamily, though such a characteristic is only inferred in Adeliinae based on morphological similarities between Adeliinae and the remaining chelonines (Čapek 1971 and Nixon 1965, cited by Shaw and Huddleston 1991).

The genus *Leptodrepana* Shaw was first described in 1983 and is represented by four species (Shaw 1983). Prior to that time *Leptodrepana* species were placed in the genus *Ascogaster* Wesmael. A taxonomic study of *Ascogaster*, however, resulted in the discovery of the genus *Leptodrepana* (Shaw 1983). *Leptodrepana* is separated from *Ascogaster* based on 13 diverging morphological characters (Shaw 1983). In *Leptodrepana*, the ocellar triangle is equilateral. The occipital carina, while complete, tends to be weak dorsally. Evenly scattered minute setae are present over the eyes. The median frontal carina is often weak. The scutellar disc is generally flat and polished. The ovipositor is long, slender, and tends to be curved upward. The carapace apex is often flared above the ovipositor. In females, the flagellum often consists of 17 segments, the apical flagellomeres tend to be longer than wide, and the ventral cavity is as long as the carapace. The scutellar sulcus has 7–10 well-defined depressions. The propodeal tubercles are usually short or indistinct. The metatibial spurs are less than half the length of the metabasitarsus. And, where host associations are known, members of the genus are associated with plutellid hosts (Lepidoptera: Plutellidae). *Leptodrepana* species are distributed across Central and South America (Shaw 1997).

In contrast, *Ascogaster* has an isosceles-shaped ocellar triangle. The occipital carina is strong. The eyes are consistently glabrous. The median frontal carina is often present. The scutellar disk is bulging and often sculptured. The ovipositor tends to be short and retracted under the carapace. The carapace apex is not flared above the ovipositor. In females, the flagellum consists of 25 or more segments, the apical flagellomeres are often almost as long as wide, or shorter, in length than width and the ventral cavity is shorter than the length of the carapace. The scutellar sulcus generally bears 4–6 well-defined depressions. The propodeal tubercles are well-developed. The metatibial spurs tend to be greater than half the length of the metabasitarsus. And, hosts consist primarily of tortricids (Lepidoptera: Tortricidae).

The best characters for distinguishing between the two genera are the shape of the ocellar triangle, shape and number of flagellomeres in the female, and degree of convexity in the scutellar disc.

Despite these noticeable character differences the generic designation of *Leptodrepana* is still debated. This is largely due to the presence of a few intermediate forms of the genera in other regions. Some species examined in the Palearctic region have characters intermediate to the states mentioned above making it difficult to identify them as either *Leptodrepana* or *Ascogaster* (Tang and Marsh 1994, Achterberg 1990). Some European taxonomists accept *Leptodrepana* as a valid genus (Zettel 1990). Others do not. For example, Huddleston’s (1984) revision of the Palearctic *Ascogaster* did not address the complication created by the presence of intermediate forms; instead the work completely ignored the new genus. In 1990, the problem was discussed by Achterberg and he synonymized the genus, albeit without an adequate explanation or formal examination of the group. Although accepting Achterberg’s (1990) synonymy appears to solve the problem of chelonine morphs that fall somewhere between *Ascogaster* and *Leptodrepana*, it maintains another conceptual problem; *Ascogaster* would become a clearly paraphyletic group. A phylogenetic analysis of chelonine wasps, based on 16s rDNA, supports Shaw’s (1983) recognition of the genus *Leptodrepana* as separate from *Ascogaster* (Chen 1999). A more recent analysis by Kittel et al. (2016), based on three molecular markers (*COI*, *EF1α*, *28S*) and 37 morphological characters, recovered *Ascogaster* into two separated clades, one mostly Australian and one mostly North American. The genera *Austroascogaster* Kittel & Austin, 2014, *Leptodrepana*, and *Megascogaster* Baker, 1926 are located between the two clades, thus rendering *Ascogaster* paraphyletic. Kittel et al. propose two scenarios to solve the paraphyly: one considers *Ascogaster* *sensu lato*, i.e., to group all the clades into *Ascogaster*; the other considers *Ascogaster* *sensu stricto*, i.e., to restrict the genus to the North American clade. The last scenario provides some insight about the patterns of biogeographic divergence among the clades, recognizes documented morphological differences among the genera, and maintains the monophyly of each genus. Moreover, an eventual nomenclatural change supporting the first scenario requires a more comprehensive data set (Kittel et al. 2016).

The Cheloninae are egg-larval endoparasitoids of microlepidoptera (Shaw and Huddleston 1991). Shaw (1995, 1997) has suggested that exploiting the exposed and vulnerable egg stage of a host may be a means of simplifying the host location and oviposition process. Chelonines are koinobionts, so parasitized eggs are allowed to develop and mature during the larval stage. The third instar larva of the wasp emerges from the last larval stage of the host within the host pupation chamber (Shaw and Huddleston 1991). After emergence, the wasp larva feeds externally before spinning a cocoon and pupating while still inside the pupation chamber of the host (Shaw 1995). There has been little biological study of *Leptodrepana*. Shaw (1983) was able to associate *L. opuntiae* with a plutellid host but no other host information is available for other described *Leptodrepana* species or those treated in this paper.

The present work is a taxonomic study of Costa Rican *Leptodrepana.* Due to the unique efforts of the Malaise Network in place in Costa Rica, the country is especially well-sampled and a surprising number of *Leptodrepana*, normally rare species, have been collected (see Materials and methods).

## Materials and methods

Approximately 292 specimens were examined for this study. This number includes all identified Neotropical *Leptodrepana* available in the collections of the following institutions: Natural History Museum, London, England (**BNHM**), California Academy of Sciences, San Francisco, California (**CAS**), Instituto Nacional de Biodiversidad, Santo Domingo de Heredia, Costa Rica (**INBio**), University of Wyoming Insect Museum, Laramie, Wyoming (**UWIM**).

Most of the specimens examined in this study were collected by Malaise traps that are part of the Malaise Network. The Malaise Network is a sampling program originally supported by the Natural History Museum to aid in the inventory of Costa Rican Hymenoptera. The Malaise traps in the program were initially set up by Ian Gauld and Paul Hanson. Eventually, more than 45 sites were sampled in seven different provinces. The collected material was sorted to the family level at the Insect Museum of the University of Costa Rica. Sorted samples were then sent off to experts for further study. According to Hanson (pers. comm. 1995), the purpose of the Malaise Network is two- fold: to encourage taxonomic study and to eventually build a synoptic collection of Costa Rican Hymenoptera.

The majority of the specimens in this study were prepared at the UWIM. Prior to point mounting, specimens were immersed in 100 % ethanol for a 24-hour period. After immersion in the ethanol the specimens were briefly allowed to air-dry for a period of approximately 15 minutes. At this point, specimens were immersed in chloroform for 15 minutes (minute individuals) to 45 minutes (larger bodied individuals) and then allowed to air-dry.

In some instance, specimens were riddled with debris and required cleaning. To clean the specimen it was removed intact on the point from the pin and placed in a small mesh basket. A solution was prepared of approximately 1/2 teaspoon dry dishwasher detergent to 500 mL of warm tap water. The basket and enclosed specimen were submerged in the warm soapy solution and gently agitated for 1 minute. The specimen was allowed to stand in the solution for approximately 2 hours gently agitated 2–3 more times during this period. It was then removed from the soapy solution and immersed in three changes of warm clean water. The specimen without the point was removed from the basket and allowed to dry before being prepared in the manner given above.

A calibrated ocular micrometer was used for all measurements. For the sake of consistency, the same microscope and micrometer were used for all measurements. Images were captured with a Leica M205C stereomicroscope with digital Leica DFC295 camera kit and processed with Leica Application Suite Version 3.8.0 auto-montage software. Unless otherwise noted in the figure captions, illustrated specimens were females. Also, unless otherwise indicated, all of the material examined was collected by use of a Malaise trap, originated in Costa Rica, and is presently located at the UWIM. In addition, due to the large amount of material collected by Paul Hanson his name as collector has also been omitted except in cases of holotype data records. Therefore, unless otherwise indicated, please acknowledge P. Hanson as collector of all of the material used in this study. Collection data was recorded as it appears on the data label, although for purposes of organization some information may have been rearranged so as to follow the same sequential format of other labels.

This manuscript was developed from a master thesis written by SDD under the guidance of SRS. Authorship of the new species is attributed to SDD and SRS, with the exception of the patronymic species *L. scottshawi*, authorship of which is solely attributed to SDD. The coauthors HA and LFVA produced and arranged the images and plates for this publication, as well as contributing to the updating of the introduction, and refinement of the identification key and descriptions.

## Taxonomic characters and terminology

Costa Rican specimens can be identified to subfamily Cheloninae and genus *Leptodrepana* using the keys and characters previously provided by Shaw (1995, 1997, 2006) and Wharton et al. (2017).

The morphological characters and terminology in this study follow the works of Shaw (1983), Huddleston (1991), Tang and Marsh (1994), and Sharkey and Wharton (1997). The term precoxal sulcus is employed instead of sternaulus accordingly to Wharton (2006). Microsculpture characteristics are described in accordance to Harris (1979). Wing venation is described according to the conventions used by Sharkey and Wharton (1997).

### Head

To describe differences in head shape among species a simple width to length ratio was used. Width (HW) is expressed as the measurement of the maximum breadth of the head when the specimen is viewed anteriorly, in all cases the distance between the outer perimeters of the eyes. Length (HL) is expressed as the measurement of the maximum length of the head when the specimen is held in a lateral view, usually from the top of the ocellar triangle to the apical clypeal margin.

Female specimens in this study consistently had 17 flagellomeres with length of each segment decreasing apically. In almost all cases flagellomere length exceeded width, but in a few species length and width of the penultimate flagellomere is approximately equal and 0.5 the size of the ultimate flagellomere. Additionally, in some species the flagellum appears slightly dilated medially at flagellomeres 8–14. The number of flagellomeres in the antennae of males varied from 22–26. In males, the flagellum was always slender and long with all segments longer than wide and tapering apically.

Microsculpture and pubescence of the head is also a useful character for distinguishing between species, especially the depressed area of the frons, which may be obstructed from view by the position of the antennae. The median carina of the frons is a somewhat useful character but only insofar as it is either present or lacking.

### Mesosoma

Microsculpture of the mesopleuron was especially important. The mesopleuron was generally characterized by the type of sculpture present medially and the type of sculpture at the precoxal sulcus. In some cases a deep groove with regularly shaped depressions is present at the precoxal sulcus and in this study is termed a scrobiculate groove. This character is contrasted by a foveate band or groove at the precoxal sulcus which consists of irregularly sized pits.

Other characters include degree of organization of linear lacunose grooves in the mesoscutum, type of sculpture on the mesonotal lobes, the number of well-defined depressions in the scutellar sulcus, type of sculpture present on the lateral pronotum, and comparisons between the median and lateral propodeal tubercles.

### Wings

Wing venation patterns are very similar among the known Costa Rican species, and of little value for distinguishing species. However, fore wing color does have utility for species recognition, including the degree of pigmentation, presence of infuscate bands or obfuscate areas, and, in some cases, density of setae (see Figs 126–128). The length of the fore wing is abbreviated as FWL.

### Metasoma

Many characters of the metasoma are useful. Shape is expressed in terms of length to width ratios and both measurements are made in lateral view. Shape of the carapace apex in lateral, dorsal, and posterior views is also used to discriminate between species. The carapace apex may be rounded, squared, terminating in a single point, or terminating in two points. Microsculpture of the carapace is another useful character. Although basally areolate-rugose in all cases, species differed in microsculpture types apically. The length and width of the carapace are abbreviated as CL and CW respectively.

### Size and color

In many species body size (body length, BL) proves to be a useful character. In this study, species are considered small if range in size falls below 2.5 mm and large if range in size is above 3.0 mm. In species of intermediate size, size is not used as part of the species diagnosis. The wide gap between what are considered small and large species should serve to remove some of the problems in length ascribed to various positions of the head, and metasoma.

In this study, color patterns of specimens examined were useful characters. Shaw (1983) notes that although color may vary widely within a species, it still has diagnostic value. Indeed in examining series of material in this study, color is a fairly consistent character for distinguishing the Costa Rican species. Several *Leptodrepana* species exhibit a black/red/black body color pattern that is also shared by some Scelionidae and other insects, and may be part of a broader mimicry complex (P. Hanson, pers. comm.).

## Taxonomic part

### Genus Leptodrepana

Taxon classificationAnimaliaHymenopteraBraconidae

Shaw, 1983

#### Diagnosis.

Head wider than long and sculptured; anterior tentorial pits distinct; fronto-clypeal suture weak; apical clypeal margin rounded; mandibles bidentate with anterior tooth larger; maxillary palpi six-segmented; labial palpi four-segmented; antennae inserted high on face; scrobes carinate; ocelli prominent and arranged in an equilateral triangle; occipital carina complete and weak dorsally; female antennae with 17 flagellomeres, decreasing in length apically; male antennae with 22–26 flagellomeres, all longer than wide and tapering apically; mesosoma setose and sculptured: mesoscutum medially with roughly parallel lacunose grooves to areolate-rugose; notauli lacunose and often only visible anteriorly; mesonotal lobes varied; scutellar sulcus with 3–8 well-defined depressions; scutellar disc flat or slightly convex, polished-rugulose and punctate; anterior scutellar depression carinate, rarely foveolate; metanotum and posterior scutellar depression irregularly foveate; mesopleuron anteriorly rugose, medially varied, and indistinct to scrobiculate at precoxal sulcus; propodeum quadrate, areolate-rugose with median transverse carina raised into medial and lateral flanges; metatibial spurs shorter than half metabasitarsus length; wings setose, hyaline or infuscate; costa broken at base of stigma; three submarginal cells present in fore wing; veins RS+Mb, and r-m spectral; hind wing venation weak; metasoma dorsally setose and sculptured; tergites 1–3 fused to form a rigid carapace without transverse sutures, basally areolate-rugose; apically often flared above ovipositor; ventral cavity approximately the same length as carapace; ovipositor long slender and curved upwards; body color yellow, yellowish orange, orange, orangish brown, dark brown, brownish black, and black; body length 1.7–3.8 mm.

#### Remarks.

Twenty-four new species of *Leptodrepana* are recorded from Costa Rica. Examinations of these species reveal some variation from the characters originally used by Shaw (1983) to separate the two genera. Shaw’s (1983) diagnosis, based on North American species, states that the number of flagellomeres in females is sometimes reduced to 17, the scutellar sulcus has 7–10 depressions, and propodeal tubercles are usually short or indistinct. In Costa Rican *Leptodrepana*, the females consistently have 17 flagellomeres. The scutellar sulcus has anywhere from 3 to 8 depressions and the propodeal tubercles are often distinct, although sometimes the median flanges are much reduced in comparison to the lateral flanges. Reduction of the flagellum to just 17 flagellomeres is a character unique to only two of the N. American species described by Shaw (1983): *L. opuntiae*, and *L. oriens*. As all female Costa Rican *Leptodrepana* have 17 flagellomeres, this may be a synapomorphic character tying all Costa Rican *Leptodrepana* to *L. opuntiae* and *L. oriens*.

There is only one record of *Ascogaster* in the neotropics, *A. bugabensis* Cameron. This specimen was borrowed from the Natural History Museum (London) and upon examination proved to be a helconine in the genus *Urosigalphus* (Shaw and Dadelahi, 2002). However, based on our own vast experience sorting Costa Rican braconids, *Ascogaster* and *Leptodrepana* are easily distinguished with few, if any, of the intermediate forms that are (rarely) found in the Palearctic. Our examination of undescribed Costa Rican *Ascogaster* species found none with the number of flagellomeres reduced to 17.

#### Key to females of Costa Rican *Leptodrepana* species

**Table:** 

1	Carapace apex appearing broadly or narrowly rounded in dorsal and posterior views (Figs 16, 18, 26, 28, 33, 59, 79, 123). Sometimes carapace ventral border slightly concave in posterior view (Figs 31, 109), but protuberances or tubercles absent	**2**
–	Carapace apex terminating in one (Figs 10, 11, 84, 86, 99, 101) or two points (tubercles) in dorsal and posterior view (Figs 4, 21, 23, 43, 71); apex may appear distinctly squared if tubercles are widely separated (Figs 54, 56)	**10**
2	Head, mesopleuron, and metasoma entirely black in lateral view (as in Fig. 77); legs and antennae variable	**3**
–	Head, mesopleuron, and metasoma not entirely black in lateral view, sometimes metasoma basally white-yellow (Figs 72, 116)	**4**
3	Wings with infuscate bands (Fig. 127); mesopleuron medially shiny and impunctate; antennae dark brown except for flagellomeres 2–4 (Fig. 77)	***L. rosanadana* sp. n.**
–	Wings obfuscate or at most hyaline; mesopleuron medially densely foveate; antennae uniformly brown with distal ends of flagellomeres 1–4 yellowish white (Fig. 29)	***L. eckerti* sp. n.**
4	Mesonotum yellowish orange (as in Fig. 58); mesopleuron mostly yellow to orange; propodeum black, orange, or orange and black; carapace black with basal third yellowish white or with medio-basal yellowish white patch	**5**
–	Mesosoma mostly black; carapace variable	**7**
5	Antennae tri-colored (basally yellow, medially white and distally brown/black) (Fig. 57); mesonotum and metanotum yellow-orange with brown/black propodeum; scutellar sulcus with three well defined depressions; carapace apex with distinct ridge or carina in posterior view (Fig. 59)	***L. munjuanae* sp. n.**
–	Antennae uniformly brown or with scape, pedicel and first flagellomere yellowish white; mesonotum and metanotum orange or burnt orange; propodeum black or orange sometimes bearing black patches; scutellar sulcus with five well-defined depressions; carapace without distinct ridge in posterior view	**6**
6	In lateral view, carapace no more than 3× as long as wide (Fig. 17); mesopleuron orange anteriorly and black posteriorly; propodeum black; carapace black with basal third yellowish white; fore leg, middle leg, and hind leg mostly yellowish white but hind leg with apical portions of coxa, tibia, and femur dark brown (Fig. 14)	***L. conda* sp. n.**
–	In lateral view, carapace approximately 4× as long as wide (Fig. 27); mesopleuron orange or burnt orange; propodeum orange sometimes with small brownish black area over medial flanges; carapace black sometimes with media-basal yellowish white patch between dorsal carinae but not extending to lateral margin of carapace; fore leg, middle leg and hind leg mostly yellow to yellowish white, femur of hind leg with lateral patch of white and tibia with distinctive pattern: linear brown oval ring visible dorsally running most of tibia length (Fig. 24)	***L. demeter* sp. n.**
7	Mesopleuron medially punctate with deep regular foveate groove at precoxal sulcus; laterally pronotum smooth at propleural margin; carapace apex shiny and weakly punctate or shiny and rugulose with a transverse carina	**8**
–	Mesopleuron foveate to rugose with or without scrobiculate groove at precoxal sulcus; laterally pronotum rugose at propleural margin; carapace apex rugose to areolate-rugose and pubescent	**9**
8	Carapace apex shiny and impunctate (Figs 117–118); smoothly rounded in lateral and posterior views; scutellar sulcus with 4–5 well-defined depressions	***L. strategeri* sp. n.**
–	In posterior view, carapace apex rugulose and bearing a transverse carina (Figs 74–75); in lateral view, presence of apical carina gives carapace the appearance of terminating in a narrow point below midline; scutellar sulcus with 3 well-defined depressions	***L. ronnae* sp. n.**
9	Body size robust, more than 3.0 mm in length; coxae and trochanters of all legs yellowish white, femur, tibia, and tarsus dark brown, tibia with basal yellowish white band; carapace brown-black with basal quarter or less yellowish white in color (Figs 110, 111)	***L. soussanae* sp. n.**
–	Body size small, less than 2.5 mm in length; legs yellow or light brown; carapace black with basal third yellowish white in color (Fig. 125)	***L. thema* sp. n.**
10	Carapace apex terminating in a single point or tubercle in dorsal and posterior views (Figs 10, 11, 84, 86, 99, 101)	**11**
–	In dorsal and posterior views, carapace apex terminating in two points or tubercles (Figs 4, 21, 23, 43, 71); tubercles may be greatly separated giving carapace apex a distinctly squared appearance (Figs 54, 56) or produced into flange visible in dorsal view (Fig. 6)	**14**
11	In lateral view, carapace apex with lobe or rounded flange below or posterior to apical point (Fig. 105); carapace yellow-orange and with distinct pattern: in dorsal view, basal area yellowish white between dorsal carinae with lateral crescent shaped yellowish white patch posterior to basal third of carapace; head yellowish orange; wings smoky except for white band across parastigma, and parts of veins 1RS, RS+M, 1m-cu and 2CU-a (Fig. 128)	***L. sorayae* sp. n.**
–	In lateral view, carapace terminating in a single broad or narrow point (as in Fig. 12); carapace black, with or without yellowish white patch dorsally; head black; wings unpigmented or slightly dusky but no white band below parastigma	**12**
12	Mesosoma and metasoma entirely brownish black (Figs 97–98); carapace apex shiny and weakly punctate; body size less than 2.0 mm	***L. sohailae* sp. n.**
–	Mesosoma and metasoma not entirely blackish brown; carapace apex rugose to weakly areolate-rugose, body size greater than 2.0 mm	**13**
13	Mesosoma orange, mesoscutum with wide shallow notauli; mesopleuron orange and medially foveate, deeply foveate rows forming grooved band at precoxal sulcus; carapace black, areolate-rugose, and often with white-yellow patch baso-medially covering dorsal carinae or restricted to two patches over dorsal carinae (Figs 9, 13)	***L. atalanta* sp. n.**
–	Mesonotum and metanotum orange, mesoscutum with narrow notauli; mesopleuron orange and often dark brown near posterior margin, foveate medially with deep regularly foveate groove at precoxal sulcus; propodeum black, carapace black, areolate-rugose, and basal third yellowish white in color (Figs 83, 86)	***L. schuttei* sp. n.**
14	Apex of carapace with distinctly truncated appearance in dorsal view (Figs 54, 56); carapace black with diamond shape patch medially; antennae brown with medial yellowish white; legs appear distinctly banded in yellowish white and black (Fig. 52)	***L. lorenae* sp. n.**
–	Carapace apex with two distinct tubercles in dorsal and posterior views (Figs 4, 6, 37, 39, 49, 51, 64, 66, 69, 71); carapace color and pattern variable; antennae variable; legs not distinctly banded in yellowish white and black	**15**
15	Head, mesosoma, and most of metasoma black or blackish brown	**16**
–	Not as above; mesosoma orange or with patches of orange	**20**
16	Carapace apex with tubercles blunt so in dorsal view appear as barely projecting or tubercles fall within the apical plane of the carapace and only a protruding flange is visible (Figs 4, 6, 69, 71)	**17**
–	Carapace apex with tubercles strongly protruding so in dorsal view appear strongly arched between and in lateral view apex terminates in narrow point (Figs 43, 44, 49, 50)	**18**
17	In dorsal view, carapace apex with tubercles barely projecting and shallowly arched between (Figs 69, 71); lateral pronotum deeply foveate and rugose at propleural margin; mesopleuron rugose medially with deep foveate groove at precoxal sulcus	***L. pamelabbas* sp. n.**
–	In dorsal view, carapace apex sloping so that points appear planar in dorsal view and not protruding or a protruding flange visible (Fig. 6), in posterior view apex strongly arched (Fig. 4); lateral pronotum foveolate and smooth at propleural margin; mesopleuron punctate to foveolate at and posterior to precoxal sulcus, medially with small circular shiny impunctate area	***L. alexisae* sp. n.**
18	Mesopleuron medially shiny and impunctate; lateral propleural margin of pronotum shiny and impunctate; legs yellow (Fig. 40)	***L. hansoni* sp. n.**
–	Not with above combination of characters; mesopleuron medially foveate and/or rugose; lateral propleural margin rugose; legs variable	**19**
19	Carapace brownish black with basal third yellowish white in distinctive bi-lobed pattern (Fig. 51); small, less than 2.5 mm; in lateral view carapace at the most 3× as long as wide (Fig. 50); legs generally uniform yellow in color, sometimes hind tibia bearing faint basal band; antennae often dilated medially	***L. kimbrellae* sp. n.**
–	Carapace without distinctive bi-lobed pattern although basal quarter often with cream patches laterally below dorsal carinae (Fig. 39); body robust, more than 3.0 mm; in lateral view carapace more than 3.5× as long as wide (Fig. 38); hind and fore coxae brown, meso coxae white or yellowish white, femur and tibia of all legs dark brown with narrow white basal bands; antennae not dilated medially	***L. gauldilox* sp. n.**
20	Mesosoma orange or burnt orange but uniform in color (Fig. 87); medially corrugated area of mesoscutum arranged in distinct parallel rows; scutellar disc very flat; carapace often with cream patches baso-laterally below dorsal carinae (Fig. 91)	***L. scottshawi* sp. n.**
–	Mesosoma either brown with orange and black patches or orange with black areas; medially corrugated surface area not arranged in distinct parallel rows; scutellar disc slightly convex; if carapace with yellowish white markings then patch is medial and not lateral below dorsal carinae	**21**
21	Body size small, less than 2.5 mm; body mostly dark brown but mottled appearing lighter or darker in some areas (Fig. 92); mesoscutum with square orange patch medially; scutellar disc always darker than surrounding anterior scutellar depression; mesopleuron shiny and weakly punctate medially with deep regularly foveate band at precoxal sulcus	***L. shriekae* sp. n.**
–	Body size robust, greater than 2.5 mm, mesosoma mostly orange with black patches; mesoscutum with no orange patch medially; scutellar disc same color as surrounding scutellar depression; mesopleuron variable	**22**
22	Penultimate flagellomere almost as long as ultimate flagellomere and length greater than width; carapace entirely brownish black (Fig. 112)	***L. stasia* sp. n.**
–	Penultimate flagellomere approximately half the length of ultimate flagellomere and length equal to width; carapace variable	**23**
23	Carapace apex with tubercles widely separated (Figs 64, 66); carapace black with medial yellowish orange oval or diamond shaped patch running almost length of carapace; face rugulose-punctate; mesopleuron medially shiny and weakly punctate with shallow foveate band at precoxal sulcus; antennae uniformly brown	***L. ninae* sp. n.**
–	Carapace apex with tubercles narrowly separated (Figs 21, 23); carapace black with basal yellowish white “cape” (Fig. 23); face coarsely rugulose-punctate; mesopleuron foveate with slightly larger pits forming band at precoxal sulcus; antennae generally yellowish brown basally	***L. conleyae* sp. n.**

### Leptodrepana alexisae

Taxon classificationAnimaliaHymenopteraBraconidae

Dadelahi & Shaw sp. n.

http://zoobank.org/D2205D7C-B5A7-4D57-B008-942F0CF19E95

Figs 1–6 , 126

#### Diagnosis.

In dorsal view, the carapace apex of *L. alexisae* lacks projecting tubercles instead it has a protruding flange (Fig. 6); in posterior view, two planar points, either strongly or weakly arched between, are visible (Fig. 4). The mesopleuron has a small shiny impunctate area medially with the immediate surrounding area foveolate. The precoxal sulcus appears foveolate. In females, the flagellum is slightly dilated medially. Body mostly brownish black except basal quarter of carapace yellowish white.

**Figures 1–6.**
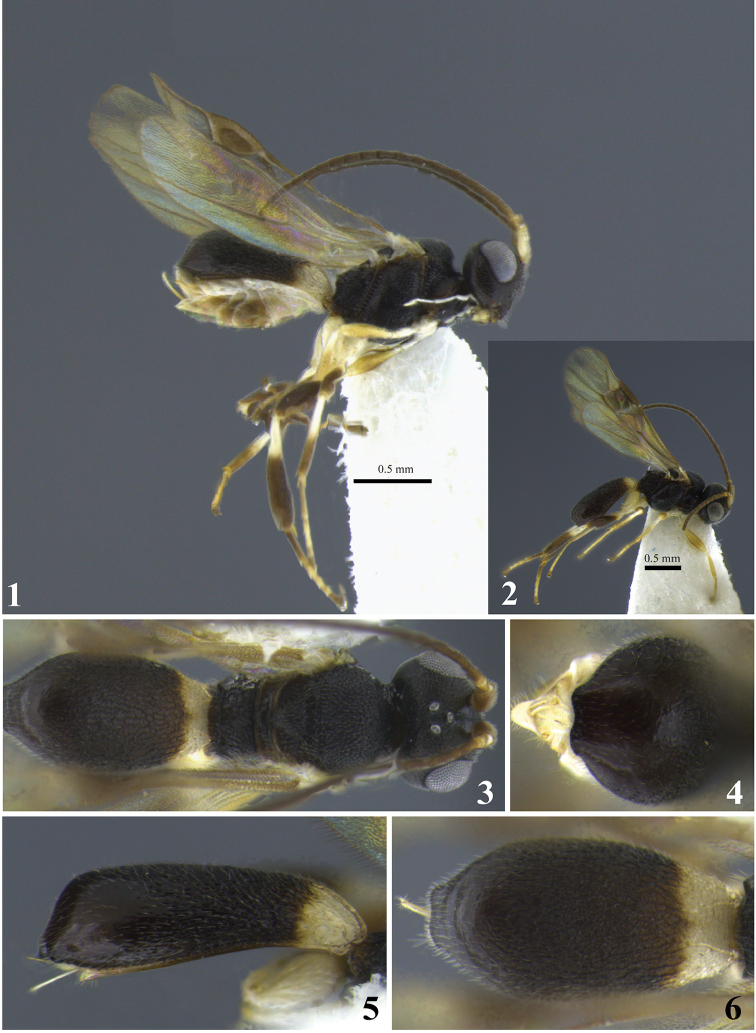
*Leptodrepana alexisae*. **1** Female habitus in lateral view **2** male habitus in lateral view **3** female habitus in dorsal view **4** metasoma in dorso-posterior view terminating in two rounded endings **5** metasoma in lateral view **6** metasoma in dorsal view.

#### Holotype female.

BL 2.07 mm; FWL 2.0 mm; CL 0.96 mm; CW 0.32 mm; CL/CW 3.

#### Description.

*Color*. Head brownish black, mandibles yellow, blackish brown apically and basally; palpi yellowish white; antennae brown with scape and pedicel yellowish white; mesosoma brownish black; legs with coxa, trochanter and trochantellus yellowish white, femur and tibia of fore leg yellow, basal half of tibia yellowish white; hind and middle legs similar to fore leg but femur and apical half of tibia dark brown; wings lightly pigmented with darker area below stigma covering apical half of 1^st^ submarginal cell and anterior portion of 2^nd^ submarginal cell; yellow/brown venation; carapace mostly black, basal quarter yellowish white.

*Head*. HW 0.63 mm; HL 0.53 mm; HW/HL 1.19; face, genae, vertex and ocellar triangle rugulose-weakly punctate; frons depressed impunctate with faint median carina; clypeus weakly punctate and apical margin rounded; occipital carina complete.

*Mesosoma*. Pronotum foveolate antero-laterally to weakly punctate at propleural margin; propleuron foveate to weakly areolate-rugose; mesoscutum medially weakly areolate-rugose, not greatly differentiated form mesonotal lobes; notauli indistinct; median and lateral mesonotal lobes foveolate-rugose; scutellar sulcus with 6–8 well-defined depressions, all longer than wide; scutellar disc sculptured similar to mesoscutum; mesopleuron anteriorly rugose foveolate, medially with small shiny impunctate area and remainder foveolate to weakly punctate, foveolate at precoxal sulcus and punctate postero-ventrally; propodeum coarsely areolate-rugose with distinct transverse carina raised into small and roughly equal medial and lateral flanges.

*Metasoma*. Carapace areolate-rugose to shiny and impunctate at apex; in posterior view apex terminates in two planar points weakly arched between; in dorsal view square rounded or truncated flange just visible below rounded carapace dorsum; in lateral view, apex terminates in sloping point below midline.

**Variation of paratype females.** Carapace apex strongly arched in posterior view, in dorsal view square rounded or truncated flange strongly protruding below rounded carapace dorsum; carapace apex with broad squared point in lateral view; mesopleuron foveate at precoxal sulcus; coxae with traces of yellowish brown; HW 0.63–0.7 mm; HL 0.53–0.6 mm; HW/HL1.17–1.19; BL 2.07–3.2 mm; FWL 2.0–2.4 mm.; CL 0.96–1.2 mm; CW 0.32–0.4 mm; CL/CW 3.0–3.25.

**Variation of paratype males.** Similar to females except antennae brown with 24 flagellomeres tapering apically; carapace apex not arched in posterior view and no protruding flange visible below rounded dorsum n dorsal view HW 0.7 mm; HL 0.58 mm; HW/HL1.21; BL 1.8 mm; FWL 1.86 mm.; CL 0.84 mm; CW 0.32 mm; CL/CW 2.62.

#### Material examined.

Holotype female: GUANACASTE, Arenales, W side Volcan Cacao, 900 m, 1988–1989 (no collector listed) [UWIM]. Paratype data: 1♀, same data except Sotobosque, 1100 m, ii.1989 (I. Gauld); 2♀, 1♂, Est. Mengo, SW Volcan Cacao, 1988–1989; 1♀, PUNTARENAS, R. B. Monteverde, San Luis 1040 m, L-N-250850-449250, xii.1992 (Z. Fuentes) [INBio, bar code1000-958034].

#### Remarks.

*Leptodrepana alexisae* is similar to and may be confused with *L. pamelabbas*. However, the following characters may be used to separate the two species. In dorsal view, the carapace apex of *L. alexisae* lacks projecting tubercles instead it has a protruding flange (Fig. 6). In posterior view, two planar points, either strongly or weakly arched between, are visible (Fig. 4). The mesopleuron has a small shiny impunctate area medially with the immediate surrounding area foveolate. The precoxal sulcus appears foveolate. In females, the flagellum is slightly dilated medially. However, in dorsal and posterior views, two small weakly protruding tubercles are visible at the carapace apex of *L. pamelabbas* (Figs 69, 71). The mesopleuron is medially rugose with wrinkles appearing somewhat parallel. At the precoxal sulcus there is a wide foveate groove. In females, the flagellum is uniform in width.

#### Etymology.

This species name is a patronym in honor of a sister of SDD, Alexis Satareh Dadelahi.

### Leptodrepana atalanta

Taxon classificationAnimaliaHymenopteraBraconidae

Dadelahi & Shaw sp. n.

http://zoobank.org/B86499A0-E4BC-4B94-9B2E-1480614F86C8

Figs 7–13

#### Diagnosis.

The carapace apex terminates in a single point; carapace black with yellowish white area baso-medially covering dorsal carinae. A wide shallow notauli and a wide band at precoxal sulcus formed by at least two shallow foveate grooves. The mesopleuron and propodeum are entirely orange, and the baso-median patch of yellowish white on the carapace does not extend to the lateral margins of the carapace.

**Figures 7–13.**
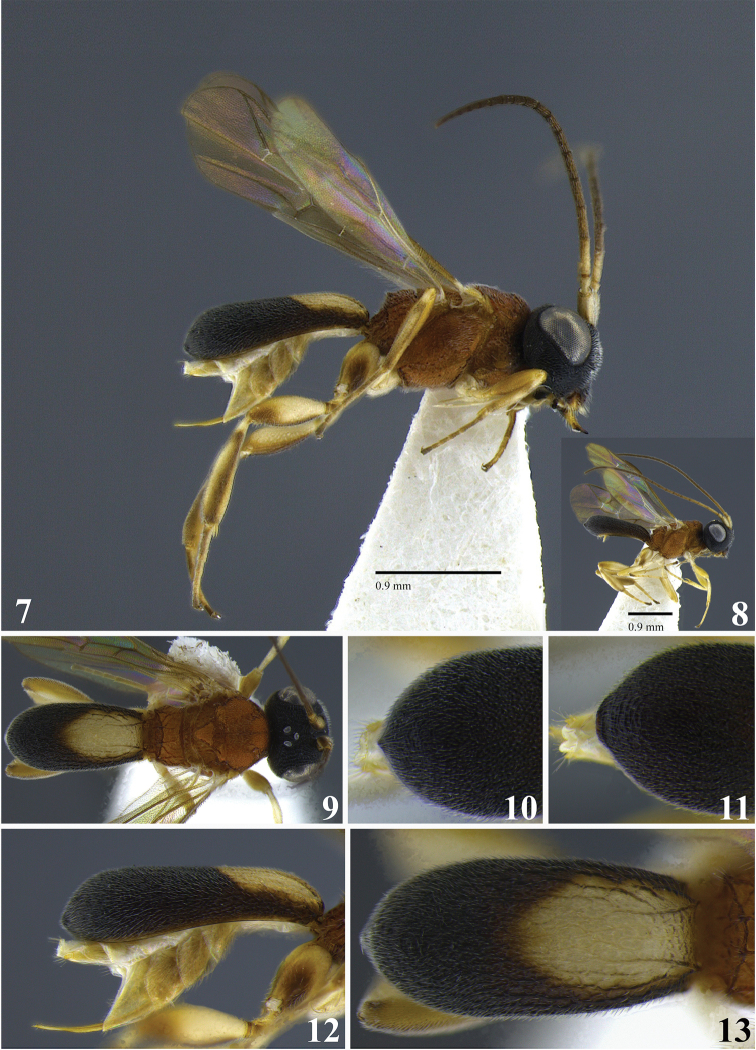
*Leptodrepana atalanta*. **7** Female habitus in lateral view **8** male habitus in lateral view **9** female habitus in dorsal view **10** metasoma in dorso-posterior view displaying a pointing end **11** metasoma in dorso-posterior view displaying a truncated end **12** metasoma in lateral view **13** metasoma in dorsal view.

#### Holotype female.

BL 3 mm; FWL 2.26 mm; CL 1.36 mm; CW 0.36 mm; CL/CW 3.7.

#### Description.

*Color*. Head black/brown, mandibles mostly yellowish brown but black/brown basally and apically; palpi yellowish white; antennae brown except scape, pedicel and first flagellomere (basal) yellowish white; mesosoma orange; legs mostly yellow, hind leg with brown areas apically on coxa and femur and white patch laterally on femur, tibia with distinct pattern: linear white ellipse ringed in brown visible dorsally running most of tibial length; wings suffused with light yellow/brown pigmentation except a small linear obfuscate area perpendicular to base of stigma in first discal cell; venation brown but veins 1M, RS+Ma, 2RS and 2M yellow or light brown; carapace black with yellowish white area baso-medially covering dorsal carinae.

*Head*. HW 0.9 mm; HL 0.78 mm; HW/HL 1.15; Face, genae, vertex and ocellar triangle rugulose-punctate; frons depressed, coarsely punctate with fine parallel lineation transverse to median carinae; clypeus weakly punctate and apical margin rounded; occipital carina complete.

*Mesosoma*. Pronotum foveate antero-laterally to weakly areolate-rugose postero-laterally; propleuron weakly areolate-rugose; mesoscutum medially with irregular parallel pitted grooves between notauli; notauli wide and shallow anteriorly; mesonotal lobes granulate-punctate; scutellar sulcus with 5 well-defined depressions, all longer than wide; scutellar disc punctate; mesopleuron anteriorly rugose and remainder deeply foveate especially at and posterior to precoxal sulcus; propodeum coarsely areolate-rugose with distinct transverse carina raised into small roughly equal medial and lateral flanges.

*Metasoma*. Carapace areolate-rugose basally graduating to coarsely rugulose-punctate at apex; apex terminating in single small point visible in dorsal and lateral views.

**Variation of paratype females.** Color: carapace with baso-medial yellowish white area not present, greatly reduced to two patches discrete to dorsal carinae or prominent covering basal half of carapace. Head: frons greatly depressed and rugose-punctate. Mesopleuron deeply foveate forming a wide pitted groove at precoxal sulcus; carapace apex rounded. HW 0.78–1 mm; HL 0.65–0.88 mm; HW/HL 1.14–1.2; BL 2.4–3.3 mm; FWL 1.86–3 mm; CL 1.12–1.6 mm; CW 0.32–0.44 mm; CL/CW 3–3.7.

**Variation of paratype males.** Similar to females except antennae with 25–26 flagellomeres tapering apically; ventral cavity distal carapace apex.

#### Material examined.

Holotype female: PUNTARENAS, Golfo Dulce 3 km SW Rincon, 10 m, iii–v.1989. (P. Hanson) [UWIM]. Paratype females: 2♀♀, GUANACASTE, Est. Pitilla, 9 km S Santa Cecilia 700 m, iv.1989 (I. Gauld); 1♀, same data except ix.1988 (I. Gauld); 1♀, same province except Arenal W side Volcan Cacao, 900 m 1988–1989; 1♀, HEREDIA, Puerto Viejo OTS, La Selva, 100 m, iv.1991; 1♀, same province Chilamate 75 m, v.1989; 1♀, same data as holotype; 1♀, same data as holotype except xii.1989–iii.1990; 3♀, same data as holotype except iii–v.1989; 1♀, same data as holotype except iii.1993; 2♀, same data as holotype except 24 km W Piedras Blancas, 200 m, vi–viii.1989; 2♂, same data except xii.1989–iii.1990; 1♂, same data except 15 km W Piedras Blancas, 100 m, xii.1990; 3♀, same province, Rd. to Rincon, 24 km W Pan. Amer. Hwy, 200 m, iii–v.1989 (P. Hanson & I. Gauld); 2♀, same province, Pen. Osa, 8 km S Rio Rincon, Coopemarti, 30 m, ii.1991; 3♀, same data except 23 km N Pto. Jimenez, La Palma, in large trees, 10 m, viii–ix.1991; 1♀, same province, P. N. Corcovado, Est. Sirena, 50 m, iv–viii.1989; 1♀, same data except 0–100 m, iii.1991 (G. Fonseca) L-S-270500,508300 [INBio]; 2♀, SAN JOSE, Ciudad Colon, 800 m, iv–v.1990 (L. Fournier).

#### Remarks.

*Leptodrepana atalanta* is similar to and may be confused with *L. schuttei*. They are similar in color and the carapace apex of both species terminates in a single point. *Leptodrepana atalanta* may be distinguished from *L. schuttei* by the presence of wide shallow notauli and a wide band at precoxal sulcus formed by at least two shallow foveate grooves. The mesopleuron and propodeum are entirely orange, and the baso-median patch of yellowish white on the carapace does not extend to the lateral margins of the carapace. The notauli of *L. schuttei* are narrow and there is a scrobiculate groove present at the precoxal sulcus. The posterior mesopleuron and the propodeum are black. The basal third of the carapace is completely yellowish white.

#### Etymology.

This species name stems from the Greek *Atalante*, one of the Argonauts noted for her fleetness of foot.

### Leptodrepana conda

Taxon classificationAnimaliaHymenopteraBraconidae

Dadelahi & Shaw sp. n.

http://zoobank.org/27B73277-272D-416C-9E3F-48A5CB2FD001

Figs 14–18

#### Diagnosis.

Rounded carapace apex. Head brownish black; mesosoma mostly orange except posterior half of mesopleuron black and propodeum black; wings suffused with light yellow/brown pigment, darker area below stigma; basal third of carapace yellowish white and apical 2/3 of carapace black/brown. Scrobiculate groove at the precoxal sulcus.

**Figures 14–18.**
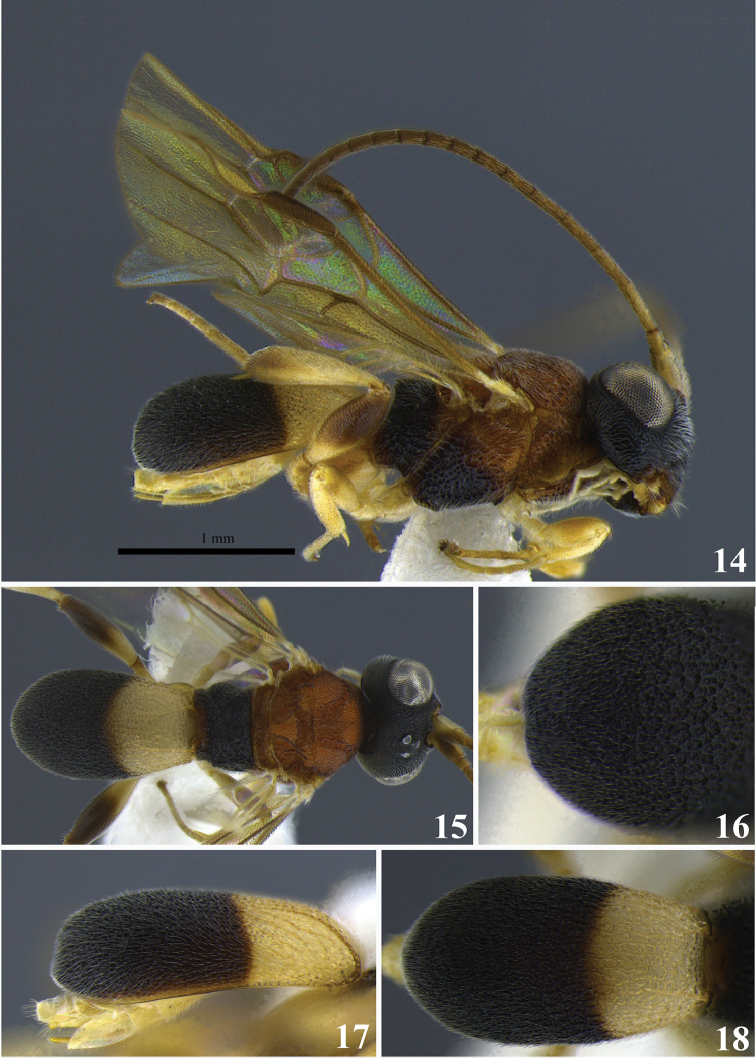
*Leptodrepana conda*. **14** Female habitus in lateral view **15** female habitus in dorsal view **16** metasoma in dorso-posterior view terminating rounded **17** metasoma in lateral view **18** metasoma in dorsal view.

#### Holotype female.

BL 3.13 mm; FWL 2.53 mm; CL 1.44 mm; CW 0.48 mm; CL/CW 3.

#### Description.

*Color*. Head brownish black, mandibles yellow, brownish black apically; apical margin of clypeus yellowish brown; palpi yellowish white; antennae brown with scape and pedicel and flagellomeres 1 and 2 (basal) yellowish white; mesosoma mostly orange except posterior half of mesopleuron black and propodeum black; middle and fore legs yellowish white; hind leg mostly yellowish white except apical portions of coxa, trochantellus, femur, and tibia brown; wings suffused with light yellow/brown pigment, darker area below stigma covering apical half of 1^st^ submarginal cell and anterior portion of 2^nd^ submarginal cell; venation yellowish brown except veins 1M and RS+M yellow; basal third of carapace yellowish white, margin sharply demarcated, and apical 2/3 of carapace black/brown.

*Head*. HW 1.0 mm; HL 0.725 mm; HW/HL 1.38; face, genae, vertex and ocellar triangle rugulose-punctate; frons depressed, weakly punctate with fine parallel lineation lateral to median carina; clypeus punctate and apical margin rounded; occipital carina complete.

*Mesosoma*. Pronotum foveate antero-laterally to impunctate at propleural margin; propleuron weakly areolate-rugose; mesoscutum medially with irregular parallel pitted grooves between notauli difficult to distinguish and appears areolate-rugose; notauli distinct and visible anteriorly; median and lateral mesonotal lobes rugose-punctate; scutellar sulcus with 5 well-defined depressions, all longer than wide; scutellar disc punctate; mesopleuron anteriorly rugose, medially deeply foveate, scrobiculate groove at precoxal sulcus, foveolate to punctate postero-ventrally; propodeum coarsely areolate-rugose with distinct transverse carina raised into small and roughly equal medial and lateral flanges.

*Metasoma*. Carapace completely areolate-rugose; in posterior and dorsal views, apex of carapace rounded.

**Variation of paratype females.** HW 0.825–1 mm; HL 0.7–0.8 mm; HW/HL 1.18–1.38; BL 2.6–3.2 mm; FWL 2.2–2.53 mm; CL 1.28–1.44 mm; CW 0.4–0.44 mm; CL/CW 2.9–3.0.

**Paratype males.** No males.

#### Material examined.

Holotype female: GUANACASTE, Cerro el Hacha, NW Volcan Orosi, 300 m, 1988 (no coll. listed) [UWIM]. Paratype data: 1♀, same data as holotype except P. N. Santa Rosa, San Emilio, tropical dry forest, 19.vi–10.vii.1995 (Dadelahi, Price & Zitani); 1♀, same data except site #6, Bosque San Emilio, 50 year old deciduous forest more or less fully shaded as possible, 300 m, 3–24.viii.1985 (I. Gauld & D. Janzen); 1♀, same data except Bosque Humedo mature evergreen dry forest more or less fully shaded as possible, 300 m, 20.xii.1986–10.i.1987.

#### Remarks.

*Leptodrepana conda* may be confused with *L. demeter* as both females are similar in color and have a rounded carapace apex. It may be distinguished from *L. demeter* by the presence of a scrobiculate groove as opposed to a wide foveate groove at the precoxal sulcus in *L. conda*. In posterior view, the carapace apex of *L. conda* is weakly areolate-rugose and evenly rounded. Whereas in posterior view, the carapace apex of *L. demeter* is rugulose-lacunose and bears a small transverse carina directly above the posterior margin of the carapace. When viewed laterally, the carapace of *L. conda* is not more than 3× as long as wide (Fig. 17). In lateral view, the carapace of *L. demeter* is more than 4× as long as wide (Fig. 27). Additionally, the propodeum of *L. conda* is blackish brown and the basal third of the carapace is completely yellowish white. The propodeum of *L. demeter* is mostly orange and the baso-median patch of yellowish white, when present, does not extend to the lateral margins of the carapace. Superficially *L. conda* also resembles the *L. atalanta* in color and size. However, the apex of *L. conda* is rounded in dorsal view, while the carapace apex of *L. atalanta* may terminate in a pointed or truncated end (Figs 10, 11).

#### Etymology.

This species name is an arbitrary arrangement of letters to form a euphonious combination.

### Leptodrepana conleyae

Taxon classificationAnimaliaHymenopteraBraconidae

Dadelahi & Shaw sp. n.

http://zoobank.org/724745CF-549A-4905-9646-37189A1A4CFB

Figs 19–23

#### Diagnosis.

The carapace apex terminates in two small tubercles weakly arched between. Head blackish brown; mesosoma mostly orange, metapleuron with black areas posteriorly; wings suffused with light yellowish brown pigment. The carapace has a baso-medial yellowish white “cape” or patch that extends laterally but not all the way to the lateral margins of the carapace, remainder of carapace blackish brown.

**Figures 19–23.**
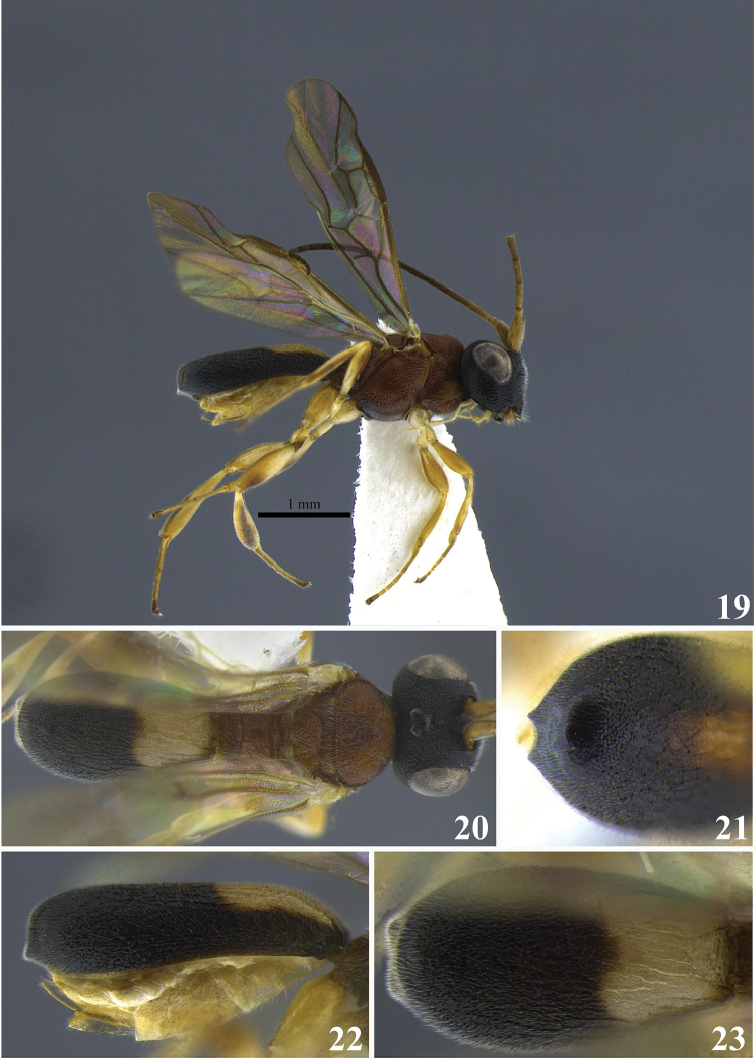
*Leptodrepana conleyae*. **19** Female habitus in lateral view **20** female habitus in dorsal view **21** metasoma in dorso-posterior view terminating in two pointing endings **22** metasoma in lateral view **23** metasoma in dorsal view.

#### Holotype female.

BL 3.7 mm; FWL 3.2 mm; CL 1.68 mm; CW 0.44 mm; CL/CW 3.8.

#### Description.

*Color*. Head blackish brown, mandibles yellow, blackish brown apically; palpi yellowish white; antennae with scape, pedicel and basal flagellomere 1 light brown and remainder of flagellum brownish black; mesosoma mostly orange, metapleuron with black areas posteriorly; fore leg yellow; hind and middle legs mostly yellow except trochantellus, apical portion of femur and laterally along length of tibia brown; wings suffused with light yellowish brown pigment; venation yellowish brown; basal third of carapace yellowish white with small median area of yellowish white extending into middle third, remainder of carapace blackish brown.

*Head*. HW 1.1 mm; HL 0.93 mm; HW/HL 1.19; face and genae, coarsely rugulose-punctate; vertex and ocellar triangle coarsely rugulose-punctate almost weakly areolate-rugose; frons depressed, coarsely rugulose-punctate with fine parallel lineation lateral to median carina; clypeus punctate and apical margin rounded; occipital carina complete; antennae with flagellomeres all longer than wide and decreasing in length apically, except penultimate flagellomere almost as long as wide and approximately half the length of ultimate flagellomere.

*Mesosoma*. Pronotum rugose-foveate antero-laterally to impunctate at propleural margin; propleuron weakly areolate-rugose; mesoscutum areolate-rugose, mesonotal lobes not differentiated from median mesoscutum; notauli indistinct; scutellar sulcus with 7 well-defined depressions, all longer than wide; scutellar disc punctate; mesopleuron anteriorly rugose, medially foveate, foveate groove at precoxal sulcus, foveolate to punctate postero-ventrally; propodeum coarsely areolate-rugose with distinct transverse carina raised into small and roughly equal medial and lateral flanges.

*Metasoma*. Carapace areolate-rugose basally to shiny and punctate at apex; in dorsal view, apex terminates in two small points visible below rounded dorsum carapace; in posterior view, apex terminates in two small points weakly arched between; and in lateral view, carapace apex terminates in narrow point below midline.

**Variation of paratype females.** HW 0.875–1.025 mm; HL 0.75–0.9 mm; HW/HL 1.12–1.2; BL 2.9–3.6 mm; FWL 2.6–3.2 mm; CL 1.36–1.6 mm; CW 0.4–0.44 mm; CL/CW 3.4–4.0.

**Paratype males.** No males.

#### Material examined.

Holotype female: PUNTARENAS, San Vito, Est. Las Alturas, 1500 m, i.1992 (P. Hanson) [UWIM]. Paratype data: 1♀, same data as holotype; 1♀, same data except xii.1991; 1♀, same data except xi.1991; 1♀, same province, R. B. Carara, Est. Queb. Bonita, 50 m, L-NL194500-469850, xii.1992 (J. C. Saborio) [INBio, barcode CR1000-900653]; 1♀, same data except i.1994 (R.M. Guzman) [INBio, barcode CR1001-940130]; 1♀, same province, Area de Conservacion Arenal, R. B. Monteverde, Est. La Casona, 1520 m, L-N-253250-449700, xii.1993, (N. G. Obando) [INBio, barcode CR1001-866000]; 1♀, same province, Coto Brus, Est. Las Alturas 1500 m, L-S-322500-591300, xii.1991 (M. A. Zumbado) [INBio, barcode CR1000-487177].

#### Remarks.

*Leptodrepana conleyae* is superficially similar to *L. ninae* but may be distinguished by the following combination of characters. In dorsal and posterior views the carapace apex of *L. conleyae* terminates in two small tubercles weakly arched between (Figs 21, 23). The propleural margin of the pronotum is shiny and weakly punctate. The mesopleuron is foveate medially and has a foveate groove at the precoxal sulcus. The scutellar sulcus has seven well-defined depressions. The carapace has a baso-medial yellowish white “cape” or patch that extends laterally but not all the way to the lateral margins of the carapace. In *L. ninae*, the carapace apex terminates in two sharp tubercles that are deeply arched between (Fig. 64). The propleural margin of the pronotum is rugose. The mesopleuron is medially shiny and weakly punctate with a shallow foveate band at the precoxal sulcus.

The scutellar sulcus has six well-defined depressions. Dorsally the carapace bears a large oval area of orangish brown.

#### Etymology.

This species is a patronym for Jennifer Katherine Conley, in appreciation for her support, friendship, and ability to argue coherently on any topic for any length of time.

### Leptodrepana demeter

Taxon classificationAnimaliaHymenopteraBraconidae

Dadelahi & Shaw sp. n.

http://zoobank.org/9B11025F-06FB-4E29-9626-90E5E45D537A

Figs 24–28

#### Diagnosis.

Rounded carapace apex. In posterior view, the carapace apex is rugulose-lacunose and bears a small transverse carina directly above the posterior margin of the carapace. In lateral view the carapace is more than 4× as long as wide. Head black-brown; mesosoma mostly orange with some brownish black areas; carapace black with yellowish white elongate oval area baso-medially between dorsal carinae. Presence of the wide foveate groove at the precoxal sulcus.

**Figures 24–28.**
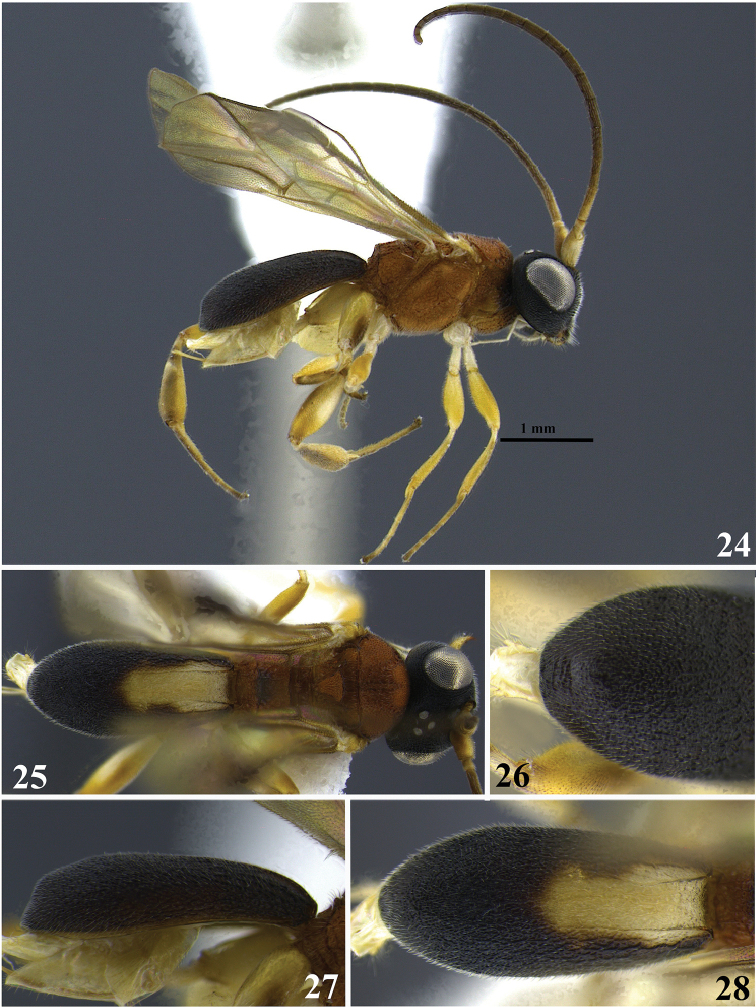
*Leptodrepana demeter*. **24** Female habitus in lateral view **25** female habitus in dorsal view **26** metasoma in dorso-posterior view terminating rounded **27** metasoma in lateral view **28** metasoma in dorsal view.

#### Holotype female.

BL 3.4 mm; FWL 3.06 mm; CL 1.92 mm; CW 0.4 mm; CL/CW 4.8.

#### Description.

*Color*. Head black/brown, mandibles mostly yellowish brown but black/brown basally and apically; palpi yellowish white; antennae brown except scape, pedicel and base of first flagellomere yellowish white; mesosoma mostly orange with some brownish black areas on median pronotum (visible laterally), mesopleuron postero-ventrally and over the median flanges of the propodeal carina; legs mostly yellow, hind leg with brown areas apically on coxa and femur and white patch laterally on femur, tibia with distinct pattern: linear white ellipse ringed in brown visible dorsally running most of tibial length; wings suffused with light yellow/brown pigmentation; venation brown but veins 1M, RS+Ma, 2RS and 2M yellow or light brown; carapace black with yellowish white elongate oval area baso-medially between dorsal carinae.

*Head*. HW 0.95 mm; HL 0.83 mm; HW/HL 1.15; face, genae, vertex and ocellar triangle rugulose-punctate; frons depressed, impunctate with fine parallel lineation transverse to median carinae; clypeus rugulose-punctate and apical margin rounded; occipital carina complete.

*Mesosoma*. Pronotum foveate antero-laterally to weakly areolate-rugose to smooth postero-laterally; propleuron weakly areolate-rugose; mesoscutum medially with 2–3 irregular parallel pitted grooves between notauli; notauli distinct and visible anteriorly; mesonotal lobes granulate-punctate; scutellar sulcus with five well-defined depressions, all longer than wide; scutellar disc punctate; mesopleuron anteriorly rugose and medially foveate; precoxal sulcus with a wide foveate groove; propodeum coarsely areolate-rugose with distinct transverse carina raised into small roughly equal medial and lateral flanges.

*Metasoma*. Carapace areolate-rugose basally graduating to rugulose-lacunose at apex; in dorsal and lateral views, carapace apex rounded; in posterior view, apex rounded and bearing a small transverse carina directly above the posterior margin of the carapace.

**Variation of paratype females.** Color: apical margin of clypeus brownish orange; carapace small baso-medial area of brownish orange; HW 0.85–0.88 mm; HL 0.68–0.73 mm; HW/HL 1.2–1.26; BL 2.86 mm; FWL 2.33 mm; CL 1.4 mm; CW 0.32 mm; CL/CW 4.37.

**Paratype males.** No males.

#### Material examined.

Holotype female: GUANACASTE, Arenales W. side Volcan Cacao, 900 m, 1988–1989 (P. Hanson) [UWIM]. Paratype females: 1♀, same province, Est. Pitilla, 9 km S Santa Cecilia, 700 m, v.1989 (I. Gauld); 1♀, same data except iv.1989.

#### Remarks.

*Leptodrepana demeter* may be confused with *L. conda* as both females are similar in color and have a rounded carapace apex. It may be distinguished from *L. conda* by presence of the wide foveate groove at the precoxal sulcus as opposed to a scrobiculate groove in *L. conda*. In posterior view, the carapace apex of *L. demeter* is rugulose-lacunose and bears a small transverse carina directly above the posterior margin of the carapace. Whereas in posterior view, the carapace apex of *L. conda* is weakly areolate-rugose. When viewed laterally, the carapace of *L. demeter* is more than 4× as long as wide. In *L. conda* the carapace is not more than 3× as long as wide. Additionally the propodeum of *L. demeter* is mostly orange and the baso-median patch of yellowish white, when present, does not extend to the lateral margins of the carapace. The propodeum of *L. conda* is blackish brown and the basal third of the carapace is completely yellowish white.

Superficially *L. demeter* also resembles *L. atalanta* in color and size. However, the apex of *L. demeter* is rounded in dorsal view (Figs 26, 28), while the carapace apex of *L. atalanta* may terminate in a single pointed or truncated end (Fig. 10, 11).

#### Etymology.

This species name stems from the Greek *Demeter* representing the goddess of agriculture, sister of Zeus, and mother of Persephone.

### Leptodrepana eckerti

Taxon classificationAnimaliaHymenopteraBraconidae

Dadelahi & Shaw sp. n.

http://zoobank.org/9EA5B13D-0A23-463F-802F-4CCCF74D4AF4

Figs 29–33

#### Diagnosis.

Robust body, more than 3.0 mm. In dorsal, lateral and posterior views, the carapace apex is rounded; in posterior view, the ventral margin of the carapace is shallowly arched. The mesopleuron medially is densely foveate and a wide foveate band is present at the precoxal sulcus. The body is mostly blackish brown except for some brownish orange on mesonotum.

**Figures 29–33.**
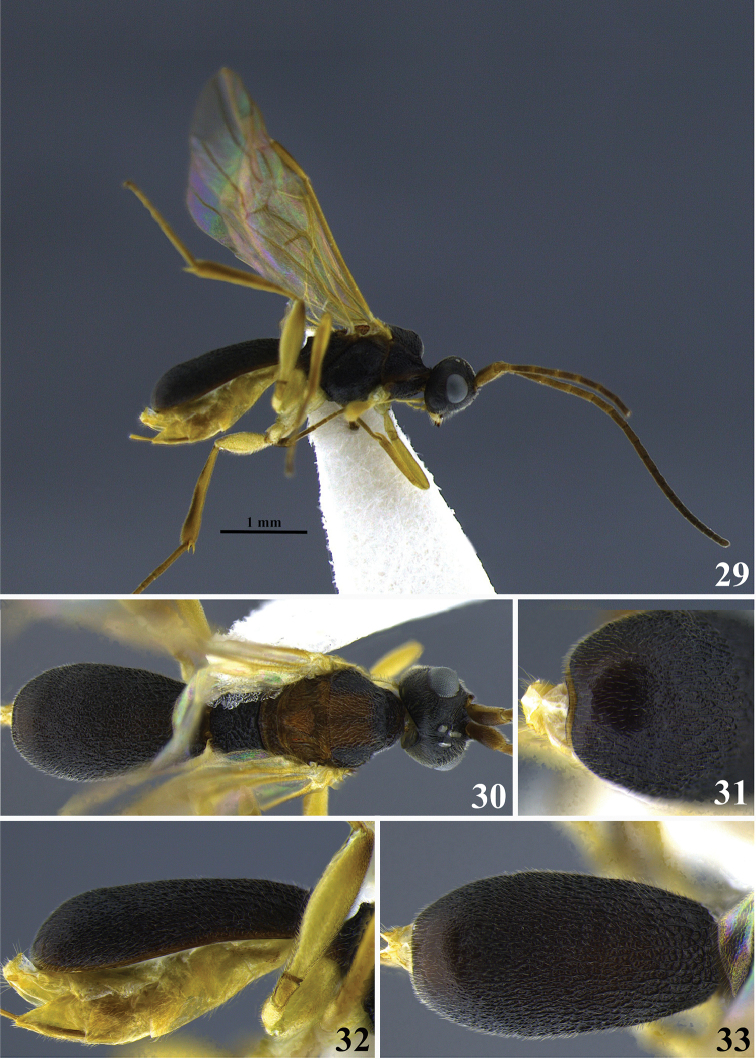
*Leptodrepana eckerti*. **29** Female habitus in lateral view **30** female habitus in dorsal view **31** metasoma in dorso-posterior view displaying a ventral, median depression **32** metasoma in lateral view **33** metasoma in dorsal view.

#### Holotype female.

BL 3.8 mm; FWL 3.8 mm; CL 1.88 mm; CW 0.52 mm; CL/CW 3.61.

#### Description.

*Color*. Head blackish brown, mandibles yellow, blackish brown apically; palpi yellowish white; antennae brown, distal ends of flagellomeres 1–4 yellowish brown, scape and pedicel yellowish brown; mesosoma mostly brownish black, mesoscutum with medial orangish brown square patch, scutellum and metanotum orange/brown; legs with coxa, trochanter and trochantellus yellowish white, femur and tibia of fore and middle leg yellowish brown, hind leg femur and tibia brown but femur yellowish white ventrally; wings lightly pigmented with yellowish brown venation; carapace blackish brown.

*Head*. HW 0.95 mm; HL 0.75 mm; HW/HL1.26; face, genae, vertex and ocellar triangle coarsely rugulose-punctate; frons depressed rugulose-punctate with parallel lineation lateral to median carina; clypeus punctate and apical margin rounded; occipital carina complete.

*Mesosoma*. Pronotum foveate antero-laterally to foveolate-rugose at propleural margin; propleuron weakly areolate-rugose; mesoscutum medially areolate-rugose; notauli indistinct; median mesonotal lobe foveolate-granulate and lateral mesonotal lobes foveolate-rugose; scutellar sulcus with five well-defined depressions, all longer than wide; scutellar disc rugose-punctate; mesopleuron anteriorly rugose foveolate, medially densely foveate, wide foveate band at precoxal sulcus, and foveolate-punctate postero-ventrally; propodeum coarsely areolate-rugose with distinct transverse carina raised into medial and lateral flanges, medial flanges greatly reduced, lateral flanges at least twice the size of median flanges.

*Metasoma*. Carapace areolate-rugose to punctate at apex; in posterior, lateral and dorsal views, apex rounded.

**Variation of paratype females.** Mesoscutum medially only orange at transcutal articulation, and scutellar sulcus; metanotum black; scutellar sulcus with 5–6 well-defined depressions; HW 0.88–0.95 mm; HL 0.75 mm; HW/HL 1.17–1.27; BL 3.5–3.8 mm; FWL 3.3–3.8 mm; CL 1.68–1.88 mm; CW 0.48–0.52 mm; CL/CW 3.3–3.61.

**Paratype males.** No males.

#### Material examined.

Holotype female: SAN JOSE, Cerro de la Muerte, 19 km S 3W Empalme, 2600 m, vi–viii.1993 (P. Hanson) [UWIM]. Paratype data: 3♀, same data except 26 km N San Isidro, 2100 m, ii–v.1991.

#### Remarks.

*Leptodrepana eckerti* may be easily distinguished from other Costa Rican species. The body is robust, more than 3.0 mm. In dorsal, lateral, and posterior views, the carapace apex is rounded (Figs 32, 33). In posterior view, the ventral margin of the carapace is shallowly arched (Fig. 31). The carapace is punctate at the apex. The mesopleuron medially is densely foveate and a wide foveate band is present at the precoxal sulcus. The scutellar sulcus has 6 well-defined depressions. The medial flanges of the propodeal carina are greatly reduced so that the lateral flanges appear at least twice as large as the medial flanges. In color, the body is mostly blackish brown except for some brownish orange on mesonotum.

#### Etymology.

This species name is a patronym in honor of Jeffery Alan Eckert in thanks for all his mental and financial support.

### Leptodrepana gauldilox

Taxon classificationAnimaliaHymenopteraBraconidae

Dadelahi & Shaw sp. n.

http://zoobank.org/C3BE4A50-9478-49BF-8C0E-6FD70669B3E2

Figs 34–39

#### Diagnosis.

Body large, more than3.0 mm, and in lateral view the carapace is more than 3.5 times as long as wide. Body mostly brownish black except yellowish white patches baso-laterally below dorsal carinae.

**Figures 34–39.**
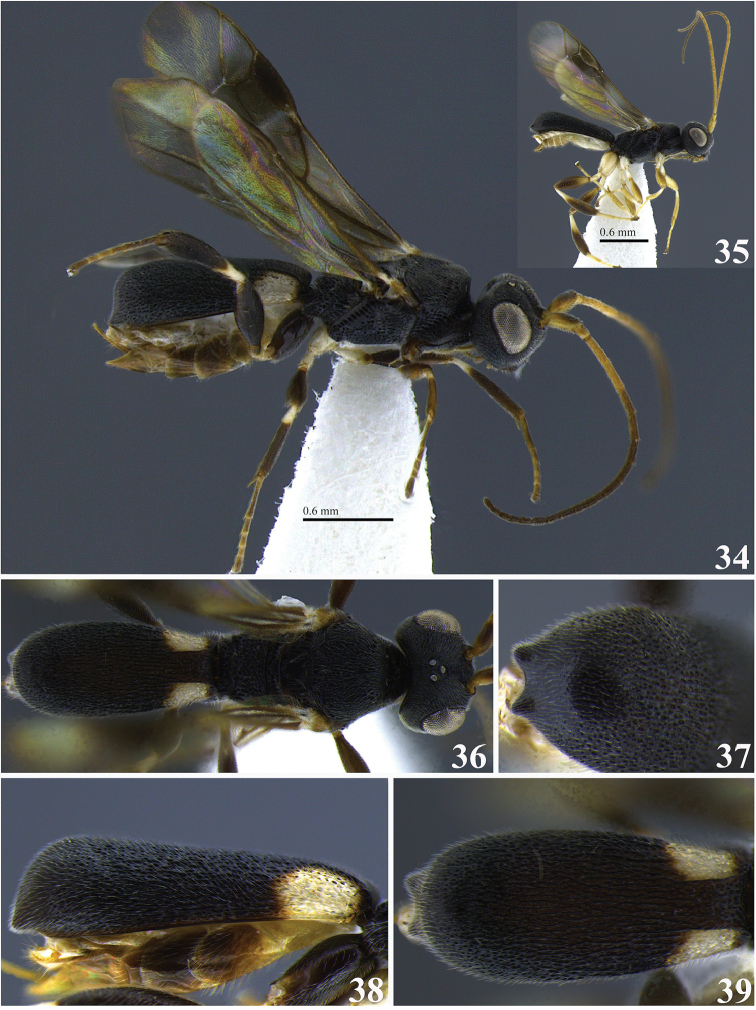
*Leptodrepana gauldilox*. **34** Female habitus in lateral view **35** male habitus in lateral view **36** female habitus in dorsal view **37** metasoma in dorso-posterior view with a couple of pointing projections **38** metasoma in lateral view **39** metasoma in dorsal view.

#### Holotype female.

BL 3.47 mm; FWL 3.0 mm; CL 1.6 mm; CW 0.44 mm; CL/CW 3.63.

#### Description.

*Color*. Head brownish black, mandibles yellow, blackish brown apically and basally; palpi yellowish white; antennae darkest apically (black) with scape, pedicel, and flagellomeres 1–5 (basal) yellow; mesosoma brownish black; hind leg: coxa dark brown, trochanter and trochantellus yellowish white, femur and tibia dark brown, tibia with narrow yellowish white basal band; fore and middle legs same as hind leg except middle coxa yellowish white; wings mostly suffused with brown pigment especially apical half of 1^st^ Submarginal, 2^nd^ Submarginal, and basal most portion of marginal cell, obfuscate linear area running from base of stigma to slightly beyond vein RS+Mb; brown venation; carapace blackish brown with yellowish white patches baso-laterally below dorsal carinae.

*Head*. HW 0.95 mm; HL 0.7 mm; HW/HL 1.36; face, genae, vertex and ocellar triangle rugulose-punctate; frons depressed rugulose-punctate, median carina present; clypeus punctate and apical margin rounded; occipital carina complete.

*Mesosoma*. Pronotum foveolate antero-laterally to rugose at propleural margin; propleuron weakly areolate-rugose; mesoscutum medially with distinct regular parallel pitted grooves between notauli; notauli narrow and visible anteriorly; median mesonotal lobe coarsely punctate and lateral mesonotal lobes rugulose-punctate; scutellar sulcus with 7 well-defined depressions, all longer than wide; scutellar disc punctate; mesopleuron anteriorly rugose, medially and at precoxal sulcus evenly foveate, foveate to weakly punctate postero-ventrally; propodeum coarsely areolate-rugose with distinct transverse carina raised into small and roughly equal medial and lateral flanges.

*Metasoma*. Carapace areolate-rugose to shiny punctate at apex; in dorsal and posterior views, apex terminating in two points widely separated; in lateral view, carapace terminates in narrow point below midline.

**Variation of paratype females.** Antennae with scape partly brown and only flagellomeres 1 and 2 yellow remainder of flagellum brown; HW 0.8–0.95 mm; HL 0.55–0.68 mm; HW/HL 1.29–1.32; BL 3.4–3.47 mm; FWL 2.6–3.0 mm; CL 1.4-1.6 mm; CW 0.32–0.44 mm; CL/CW 3.63–4.38.

**Variation of paratype males.** Similar to females except antennae with 25 flagellomeres, all longer than wide and tapering apically; scape, pedicel and flagellum yellowish white; mesopleuron sometimes medially shiny and impunctate, carapace sometimes completely black; all legs with coxa, trochanter, and trochantellus yellowish white; HW 0.73–0.8 mm; HL 0.5–0.65 mm; HW/HL 1.23–1.45; BL 2.33 mm; FWL 2.2–2.53 mm; CL 1.32–1.4 mm; CW 0.32 mm; CL/CW 4.13–4.75.

#### Material examined.

Holotype female: SAN JOSE, Zurqui de Moravia, 1600 m, ix–x.1990 (P. Hanson) [UWIM]. Paratype data: 1♀, 1♂, ALAJUELA, Area Conservation de Arenal, Est. San Ramon, in vegetation on sendero W. F. 5.vi–15.vii.1998 (Zitani, Dadelahi, Krenzelok & Fenoff); 1♀, GUANACASTE, Est. Pitilla, 9 km S. Santa Cecilia, 700 m, iv.1989 (I. Gauld); 1♂, HEREDIA, 3 km S. Puerto Viejo OTS La Selva, 100 m, xii.1992; 1♀, same as holotype except vi.1990; 1♀, same as holotype except vi.1992.

#### Remarks.

Superficially *L. gauldilox* is most similar to *L. kimbrellae* but upon closer examination they are easily distinguished from one another. *Leptodrepana gauldilox* is large, more than 3.0 mm, and in lateral view the carapace is more than 3.5 times as long as wide. Conversely, *L. kimbrellae* is small, less than 2.5 mm, and in lateral view the carapace is no more than 3 times as long as wide.

#### Etymology.

This species name is an arbitrary arrangement of letters to form a euphonious combination.

### Leptodrepana hansoni

Taxon classificationAnimaliaHymenopteraBraconidae

Dadelahi & Shaw sp. n.

http://zoobank.org/CABF0128-B4B0-4FF9-9644-A20C4157B4BC

Figs 40–45

#### Diagnosis.

The carapace apex terminates in 2 points. Body brownish black and wings lightly pigmented. Shiny impunctate area medially on the mesopleuron, and presence of four well-defined depressions in the scutellar sulcus.

**Figures 40–45.**
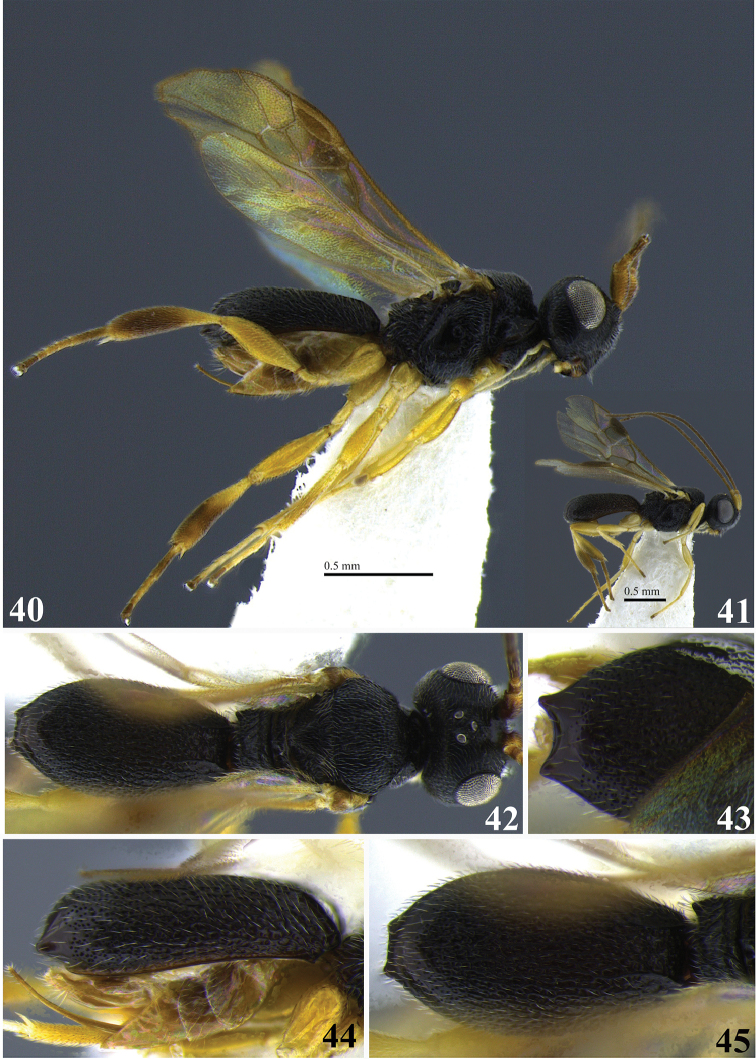
*Leptodrepana hansoni*. **40** Female habitus in lateral view **41** male habitus in lateral view **42** female habitus in dorsal view **43** metasoma in dorso-posterior view with a couple of pointing projections **44** metasoma in lateral view **45** metasoma in dorsal view.

#### Holotype female.

BL 1.87 mm; FWL 1.47 mm; CL 0.84 mm; CW 0.32 mm; CL/CW 2.63.

#### Description.

*Color*. Head brownish black, mandibles yellow, brownish black apically and basally; palpi yellowish white; antennae brown scape, pedicel, and first flagellomere (basal) yellowish brown; mesosoma brownish black; legs yellowish brown except tibia of hind leg brown; wings lightly pigmented with yellowish brown venation; carapace brownish black.

*Head*. HW 0.575 mm; HL 0.375 mm; HW/HL1.53; face finely punctuate; genae, vertex and ocellar triangle rugulose-punctate; frons depressed, weakly punctate and median carina present; clypeus punctate and apical margin rounded; occipital carina complete; antennae with flagellum dilated medially so that flagellomeres 8–14 (basal) appear almost as long as wide, remaining flagellomeres decreasing in length apically.

*Mesosoma*. Pronotum foveolate antero-laterally to shiny and impunctate at propleural margin; propleuron areolate-rugose; mesoscutum medially with irregular pitted grooves between notauli so that area appears areolate-rugose; notauli narrow and visible anteriorly; median and lateral mesonotal lobes rugose-coarsely punctate; scutellar sulcus with four well-defined depressions, all longer than wide; scutellar disc punctate; mesopleuron anteriorly rugose foveolate, medially shiny and impunctate, narrow scrobiculate groove at precoxal sulcus, weakly punctate to impunctate postero-ventrally; propodeum coarsely areolate-rugose with distinct transverse carina raised into small and roughly equal medial and lateral flanges.

*Metasoma*. Carapace areolate-rugose to shiny punctate at apex; in posterior and dorsal views, apex with 2 tubercles; in lateral view, apex terminating in narrow point below midline.

**Paratype females.** No paratype females.

**Variation of paratype males.** Similar to female except antennae with 22 flagellomeres all longer than wide and tapering apically; propleural margin of pronotum rugose; mesoscutum coarsely punctate with medial area not sculpturally differentiated from mesonotal lobes; notauli indistinct; carapace apex rounded; HW 0.63 mm; HL 0.46 mm; HW/HL 1.36; BL 2.13 mm; FWL 1.87 mm; CL 0.96 mm; CW 0.32 mm; CL/CW 3.

#### Material examined.

Holotype female: PUNTARENAS, R. F. Golfo Dulce, 3 km SW Rincon, 10 m, ii.1993 (P. Hanson) [UWIM]. Paratype data: 1♂, SAN JOSE, Ciudad Colon 800 m, iv-v.1990 (L. Fournier).

#### Remarks.

*Leptodrepana hansoni* is superficially similar to *L. kimbrellae*. They are both similar in size, color, and the carapace apex terminates in two points. It may be distinguished from *L. kimbrellae* by the shiny impunctate area medially on the mesopleuron and the presence of four well-defined depressions in the scutellar sulcus. Additionally, the carapace of *L. hansoni* is entirely brownish black. The mesopleuron of *L. kimbrellae* is deeply foveate medially. The scutellar sulcus has six well-defined depressions. The posterior margin of the basal yellowish white patch is usually deeply notched.

#### Etymology.

This species name is a patronym for Professor Paul Hanson in appreciation for his extensive efforts in collecting braconids of all shapes and sizes.

### Leptodrepana kimbrellae

Taxon classificationAnimaliaHymenopteraBraconidae

Dadelahi sp. n.

http://zoobank.org/A98E7A29-CAFA-45F2-A26B-89EAF02ACD9D

Figs 46–51

#### Diagnosis.

In posterior view apex terminating in two points deeply arched between; in dorsal view, apex terminates in two points visible below rounded carapace dorsum. The mesopleuron is deeply foveate medially. The scutellar sulcus has 6 well-defined depressions. The posterior margin of the basal yellowish white patch on the carapace is usually deeply notched. Body mostly blackish brown but carapace with basal third yellowish white, posterior margin of this area is medially deeply notched giving the basal area a bi-lobed appearance.

**Figures 46–51.**
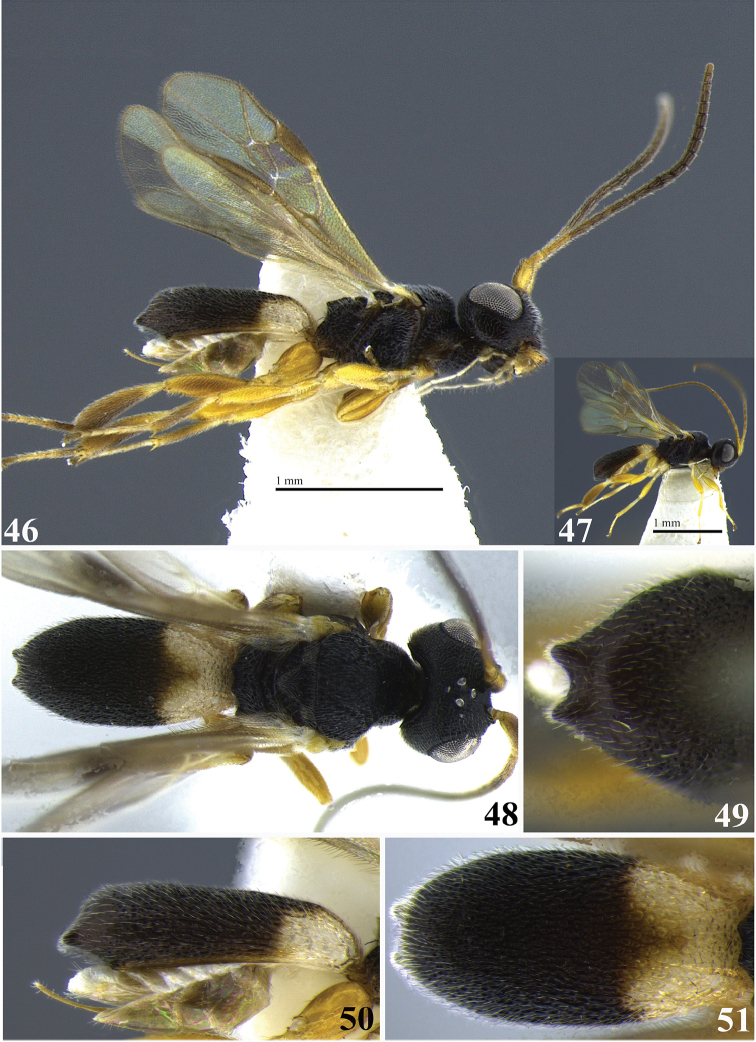
*Leptodrepana kimbrellae*. **46** Female habitus in lateral view **47** male habitus in lateral view **48** female habitus in dorsal view **49** metasoma in dorso-posterior view with a couple of pointing projections **50** metasoma in lateral view **51** metasoma in dorsal view.

#### Holotype female.

BL 2.46 mm; FWL 2.06 mm; CL 1.16 mm; CW 0.4 mm; CL/CW 2.9.

#### Description.

*Color*. Head blackish brown, mandibles mostly yellowish brown but blackish brown basally and apically; palpi yellowish white; antennae brown darkest apically with scape, pedicel and first flagellomere (basal) yellow; mesosoma black; legs yellowish orange with a narrow basal band of white on tibiae of hind and middle legs; wings suffused with light brown pigmentation and appear slightly dusky with brown venation, parastigma and vein 1M lighter in color; carapace blackish brown with basal third yellowish white, posterior margin of this area is medially deeply notched giving the basal area a bi-lobed appearance.

*Head*. HW 0.75 mm; HL 0.63 mm; HW/HL 1.2; face, genae, vertex and ocellar triangle rugulose-punctate; frons depressed, coarsely punctate with fine parallel lineation to median carinae; clypeus punctate and apical margin rounded; occipital carina complete; antennae with a slightly swollen appearance medially.

*Mesosoma*. Pronotum foveate antero-laterally to weakly areolate-rugose at propleural margin; propleuron weakly areolate-rugose; mesoscutum medially with irregular parallel pitted grooves between notauli difficult to distinguish and appears areolate-rugose; notauli narrow and visible anteriorly; medial mesonotal lobe granulate-punctate, lateral mesonotal lobes rugose-coarsely punctate; scutellar sulcus with 6 well-defined depressions, all longer than wide; scutellar disc coarsely punctate; mesopleuron anteriorly rugose, medially deeply foveate especially at and posterior to precoxal sulcus; propodeum coarsely areolate-rugose with distinct transverse carina raised into small and roughly equal medial and lateral flanges.

*Metasoma*. Carapace areolate-rugose basally, graduating to shiny and weakly punctate at apex; in posterior view apex terminating in two points deeply arched between; in dorsal view, apex terminates in two points visible below rounded carapace dorsum; in lateral view, apex terminates in sloping point below midline; ventral cavity almost reaching apex.

**Variation of paratype females.** Color: clypeus orange; scape pedicel flagellum uniformly brown; legs yellowish white to orange/brown and basal tibial bands appear faint on lighter colored legs; carapace with basal yellowish white area weakly to deeply bi-lobed; parastigma and vein 1M same color as remaining venation; HW 0.63–0.78 mm; HL 0.53–0.63 mm; HW/HL 1.2–1.25; BL 1.7–2.4 mm; FWL 2.1–2.9 mm; CL 0.9–1.2 mm; CW 0.32–0.44 mm; CL/CW 2.4–2.8.

**Variation of paratype males.** Similar to females except clypeus yellow/orange; antennae with 24 flagellomeres, tapering apically; flagellum yellowish white; mesopleuron coarsely punctate medially; in posterior view, carapace apex terminating in two points shallowly arched between; ventral cavity distal carapace apex. HW 0.58–0.63 mm; HL 0.48–0.5 mm; HW/HL 1.2–1.3; BL 1.9–2.1 mm; FWL 1.7–1.9 mm; CL 1.0 mm; CW 0.32 mm; CL/CW 3.13.

#### Material examined.

Holotype female: PUNTARENAS, R. F. Golfo Dulce, 3 km SW Rincon, 10 m, vi–viii.1989 (P. Hanson) [UWIM]. Paratype females: 3♀, 1♂, same data as holotype; 1♀, GUANACASTE, P. N. Guanacaste Arenales, SW side Volcan Cacao, 900 m, ii–v.1989 (I. Gauld); 1♀, same data as holotype except ii–iii.1989 (P. Hanson & I. Gauld); 6♀, 1♂, same data as holotype except iii–v.1989; 1♀, same data as holotype except xii.1989–iii.1990; 1♀, same data as holotype except vi.1991; 1♀, same data as holotype except x.1991; 5♀, same data as holotype except ii.1993; 1♀, 1♂, same data as holotype except iii.1993; 3♀,1♂, same data as holotype except in primary forest, iv.1993; 1♂, PUNTARENAS, P. N. Corcovado, Est. Sirena, 0–100 m, i.1992 (G. Fonseca) L-S-270500,508300 [INBio]; 1♀, PUNTARENAS, San Vito Est Biol. Las Alturas, 1700 m, ii–iv.1993; 2♀, SAN JOSE, 26 km N San Isidro 2100 m, malaise in secondary growth, ii–iv.1993; 3♀, SAN JOSE, Cerro de la Muerte, 2 km W. Empalme 2300 m, vi.1995.

#### Remarks.

Superficially *Leptodrepana kimbrellae* is most similar to *L. gauldilox*. Both species are similar in color and in posterior and lateral views, have two tubercles on the carapace apex. However, upon examination they are easily distinguished from one another. *Leptodrepana kimbrellae* is small, less than 2.5 mm and in lateral view, the carapace is at the most 3 times as long as wide. *Leptodrepana gauldilox* is large, more than 3.0 mm and in lateral view, the carapace is more than 3.5 times as long as wide. *Leptodrepana kimbrellae* is also similar to *L. hansoni* in size, color, and presence of two tubercles on the carapace apex, visible in posterior and dorsal views. *Leptodrepana kimbrellae* may be easily distinguished from this species. The mesopleuron is deeply foveate medially. The scutellar sulcus has six well-defined depressions. The posterior margin of the basal yellowish white patch on the carapace is usually deeply notched. *Leptodrepana hansoni* has a shiny impunctate area medially on the mesopleuron and the scutellar sulcus has four well-defined depressions. Additionally, the carapace of *L. hansoni* is entirely brownish black.

#### Etymology.

This species name is a patronym named in honor of a sister of SDD, Sarah Kimbrell Migrants.

### Leptodrepana lorenae

Taxon classificationAnimaliaHymenopteraBraconidae

Dadelahi & Shaw sp. n.

http://zoobank.org/9BD8D7BF-964B-474E-8175-1DA02142E947

Figs 52–56

#### Diagnosis.

Large body size, more than 3.0 mm. In dorsal view, the carapace apex in females appears distinctly truncated; in posterior view, the points are visible; in lateral view the carapace is approximately 4.5× as long as wide. Head yellowish white except vertex, temple, medial area of frons and apical margin of clypeus blackish brown; lateral pronotum and propleuron yellowish white; mesonotum orange; mesopleuron blackish brown except postero-ventrally yellowish white; metanotum, metapleuron and propodeum blackish brown; wings suffused with dark brown pigment; carapace mostly black with large yellowish white diamond shaped patch covering most of dorsal surface.

**Figures 52–56.**
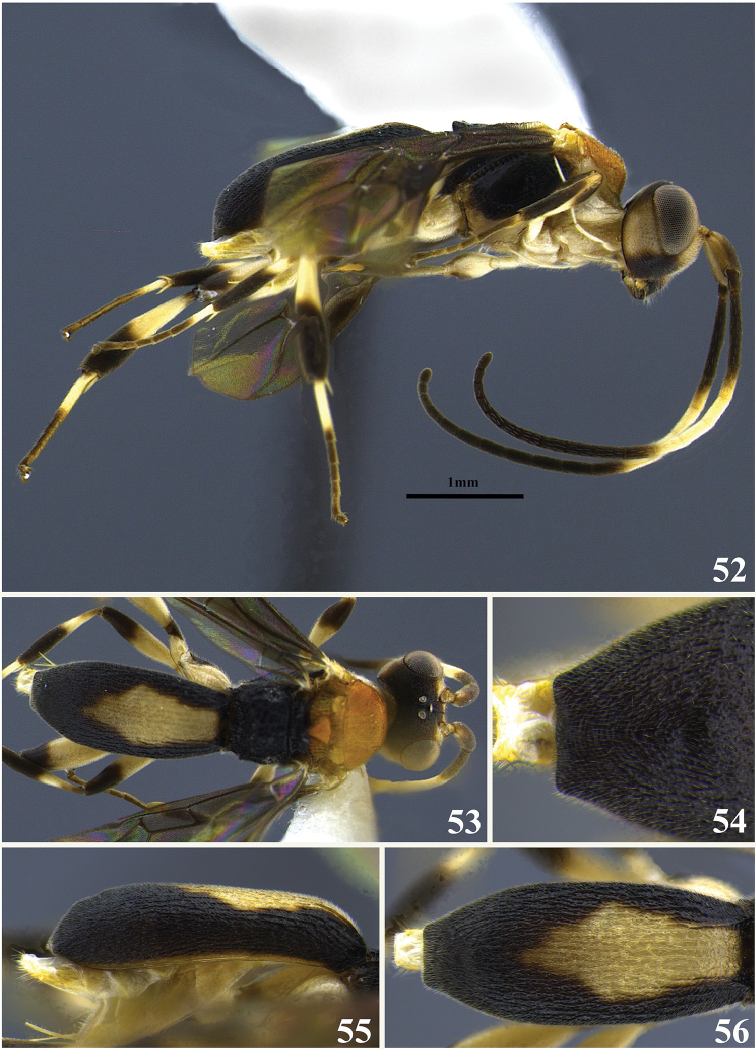
*Leptodrepana lorenae*. **52** Female habitus in lateral view **53** female habitus in dorsal view **54** metasoma in dorso-posterior view terminating truncated **55** metasoma in lateral view **56** metasoma in dorsal view.

#### Holotype female.

BL 3.6 mm; FWL 3.0 mm; CL 1.84 mm; CW 0.4 mm; CL/CW 4.6.

#### Description.

*Color*. Head yellowish white except vertex, temple, medial area of frons and apical margin of clypeus blackish brown; clypeus orange medially; mandibles yellow, blackish brown apically; palpi yellowish white; antennae with scape, pedicel and flagellomeres 1–3 brownish black, flagellomeres 3–6 yellowish white and flagellomeres 7–17 brownish black; lateral pronotum and propleuron yellowish white; mesonotum orange; mesopleuron blackish brown except postero-ventrally yellowish white; metanotum, metapleuron and propodeum blackish brown; legs appear distinctly banded in yellowish white and brownish black: fore leg yellowish white with femur basally brownish black and tibia mostly brownish black except for narrow basal band of yellowish white; middle leg similar to fore leg except tibial band bordered by brownish black basally; hind leg similar to fore and middle legs except coxa brownish black dorso-laterally, femur with wide medial band of yellowish white otherwise brownish black and tibia same pattern as femur; wings suffused with dark brown pigment; venation dark brown; carapace mostly black with large yellowish white diamond shaped patch covering most of dorsal surface.

*Head*. HW 1.05 mm; HL 0.9 mm; HW/HL 1.16; face, genae, vertex and ocellar triangle rugulose-punctate; frons depressed with fine parallel lineation lateral to median carina; clypeus punctate and apical margin rounded; occipital carina complete.

*Mesosoma*. Pronotum mostly impunctate except foveolate at mesoscutal and mesopleural margins; propleuron foveate; mesoscutum medially with numerous pitted parallel grooves; median and lateral rugose-punctate; notauli shallow and visible anteriorly; scutellar sulcus with four well-defined depressions, all longer than wide; scutellar disc punctate; mesopleuron anteriorly rugose, medially shiny and impunctate, foveate at precoxal sulcus, weakly punctate postero-ventrally; propodeum coarsely areolate-rugose with distinct transverse carina raised into medial and lateral flanges, lateral flanges at least twice the size of median flanges.

*Metasoma*. Carapace areolate-rugose basally to rugulose-punctate at apex; in dorsal view, carapace apex appears distinctly squared or truncated; in posterior view, apex terminates in two blunt points widely separated and weakly arched between, in lateral view, carapace apex terminates in blunt point below midline.

**Paratype males.** No males.

#### Material examined.

Holotype female: ALAJUELA, Est. San Ramon, 900 m, vii-xi.1995 (P. Hanson) [UWIM].

#### Remarks.

*Leptodrepana lorenae* may be easily distinguished from other Costa Rican species because of its many unique characters. The species is large, more than 3.0 mm. In dorsal view, the carapace apex in females appears distinctly truncated (Fig. 56). The square shape is due to the presence of two blunt points widely separated from one another. In posterior view, the points are visible. Additionally, in lateral view the carapace is approximately 4.5 times as long as wide (Fig. 55). On the mesosoma, the scutellar sulcus bears three well-defined depressions and the mesopleuron is medially shiny and impunctate. Coloration of the species is also quite distinct. The head yellowish white except at the vertex, temple, medial area of frons and apical margin of clypeus which are blackish brown. The antennae are brownish black but bear a yellowish white annulus at basal flagellomeres 3–6. The mesosoma is multicolored with the lateral pronotum and propleuron yellowish white. The mesonotum is orange and the remaining portions of the mesosoma are brownish black except for postero-ventrally on the mesopleuron that is yellowish white. The legs have a distinctly banded appearance, alternating sections appearing yellowish white and brownish black. The carapace mostly black with large yellowish white diamond-shaped patch.

#### Etymology.

This species name is a patronym in honor of the niece of SDD, Loren Paige Migrants, an incredible animated force of nature.

### Leptodrepana munjuanae

Taxon classificationAnimaliaHymenopteraBraconidae

Dadelahi & Shaw sp. n.

http://zoobank.org/C3645F8E-91B8-45A4-8B8C-A22F6BC10E3B

Figs 57–61

#### Diagnosis.

In dorsal view, the carapace apex is square-rounded; in posterior view, the carapace apex is rounded with a weakly arched transverse ridge or carina; in lateral view, the apex is mostly rounded but the carina is visible. Tri-colored antennae: the scape, pedicel, and flagellomeres 1–4 are yellow; the flagellomeres 4–6 are yellowish white; flagellomeres 6–17 are black. The mesoscutum medially has two wide irregular parallel-pitted grooves between the notauli. The mesopleuron is medially foveate with a wide scrobiculate groove at the precoxal sulcus. The scutellar sulcus has 3 well-defined depressions.

**Figures 57–61.**
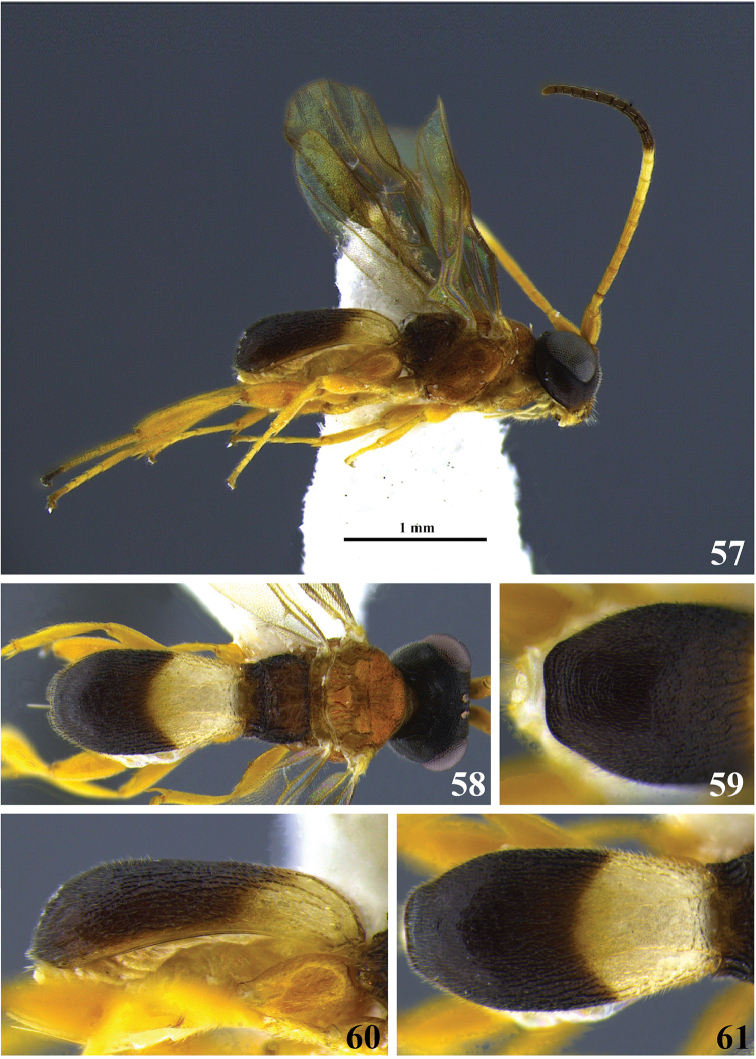
*Leptodrepana munjuanae*. **57** Female habitus in lateral view **58** female habitus in dorsal view **59** metasoma in dorso-posterior view terminating truncated **60** metasoma in lateral view **61** metasoma in dorsal view.

#### Holotype female.

BL 2.6 mm; FWL 2.13 mm; CL 1.16 mm; CW 0.44 mm; CL/CW 2.64.

#### Description.

*Color*. Head mostly brownish black, face medially yellow; clypeus yellow; mandibles yellow, blackish brown apically; apical margin of clypeus yellowish brown; palpi yellowish white; tri-colored antennae: scape, pedicel, and flagellomeres 1–4 yellow; flagellomeres 4–6 yellowish white; flagellomeres 6–17 black; mesosoma mostly yellowish orange except metapleuron and propodeum black; middle and fore leg coxae yellowish white and remainder yellow; hind leg mostly yellow except apical portions of femur, and tibia yellowish brown; wings suffused with brown pigment; basal half of carapace yellowish white, apical half of carapace blackish brown.

*Head*. HW 0.83 mm; HL 0.6 mm; HW/HL 1.38; face, genae, vertex and ocellar triangle rugulose-punctate; frons depressed, weakly punctate with fine parallel lineation lateral to median carina; clypeus punctate and apical margin rounded; occipital carina complete.

*Mesosoma*. Pronotum rugose antero-laterally to impunctate at propleural margin; propleuron weakly areolate-rugose; mesoscutum medially with two wide irregular parallel pitted grooves between notauli; notauli visible anteriorly; median and lateral mesonotal lobes rugulose-punctate; scutellar sulcus with three well-defined depressions, all longer than wide; scutellar disc punctate; mesopleuron anteriorly rugose, medially foveate, wide scrobiculate groove at precoxal sulcus, foveolate to punctate postero-ventrally; propodeum coarsely areolate-rugose with distinct transverse carina raised into small and roughly equal medial and lateral flanges.

*Metasoma*. Carapace completely areolate-rugose to rugulose-punctate at apex; in dorsal view, carapace apex square-rounded; in posterior view, apex of carapace rounded with weakly arched transverse ridge or carina; in lateral view, apex mostly rounded but carina visible.

**Variation of paratype females.** Face mostly blackish brown with yellow area reduced to clypeus and directly above fronto-clypeal suture; mesosoma mostly orangish brown; femur and tibia of hind leg not darker apically; HW 0.68–0.8 mm; HL 0.575–0.6 mm; HW/HL 1.17-1.3; BL 2.2–3.06 mm; FWL 1.8–2.53 mm; CL 1.08–1.4 mm; CW 0.28–0.4 mm; CL/CW 3.37–3.85.

**Paratype males.** No males.

#### Material examined.

Holotype female: LIMON, P. N. Tortuguera, Est. 4-esquinas, 0 m, vi–viii.1989 (Solano) [UWIM]. Paratype data: 1♀, CARTAGO, Ref. Nac. Fauna Silv., Tapanti, 1150, L-N-194000-559800, i.1992 (G. Mora & F. Quesada) [INBio, barcode CR1000-553528]; 1♀, same data except 1250 m, iii.1992, L-N-194000-560000, (G. Mora) [INBio, barcode CR1000-612020]; 1♀, PUNTARENAS, P. N. Corcovado, Est. Sirena, 50 m, iv–viii.1989 (no coll. listed); 2♀, same province R. F. Golfo Dulce, 3 km SW Rincon, primary forest, 10 m, ii.1993; 1♀, same province, Peninsula de Osa, Rancho Quemado, 200 m, L-S-292500-511000, v.1991 (F. Quesada) [INBio, barcode CR1001-294765]; 1♀, same data except v.1991, (J.C. Saborio) [INBio, barcode CR1000-594547]; 1♀, same province, Coto Brus, Est. Las Alturas, 1500 m, L-S-322500-591300, 23.iii–2.v.1992, (F. Araya) [INBio, barcode CR1000-790814].

#### Remarks.

*Leptodrepana munjuanae* has unique characters that make it easy to distinguish it from other Costa Rican species. In dorsal view, the carapace apex is square-rounded (Fig. 61). In posterior view, the carapace apex is rounded with a weakly arched transverse ridge or carina. In lateral view, the apex is mostly rounded but the carina is visible. The carapace is areolate-rugose to rugulose-punctate at the apex. The mesoscutum medially has two wide irregular parallel-pitted grooves between the notauli. The mesopleuron is medially foveate with a wide scrobiculate groove at the precoxal sulcus. The scutellar sulcus has three well-defined depressions. *Leptodrepana munjuanae* is the only Costa Rican species described that has tri-colored antennae: the scape, pedicel, and flagellomeres 1–4 are yellow; the flagellomeres 4–6 are yellowish white; flagello-meres 6–17 are black.

#### Etymology.

This species is a patronym for Munjuan Kaur, in appreciation for her friendship.

### Leptodrepana ninae

Taxon classificationAnimaliaHymenopteraBraconidae

Dadelahi & Shaw sp. n.

http://zoobank.org/40E056E0-BED8-4E24-9847-F6519B411264

Figs 62–66

#### Diagnosis.

In dorsal and posterior views, the carapace apex terminates in two sharp tubercles that are deeply arched between. Head, metanotum and propodeum brownish black; lateral pronotum and mesopleuron mostly orange. The mesopleuron is medially shiny and weakly punctate with a shallow foveate band at the precoxal sulcus. The scutellar sulcus has six well-defined depressions. Dorsally the carapace bears a large oval area of orangish brown.

**Figures 62–66.**
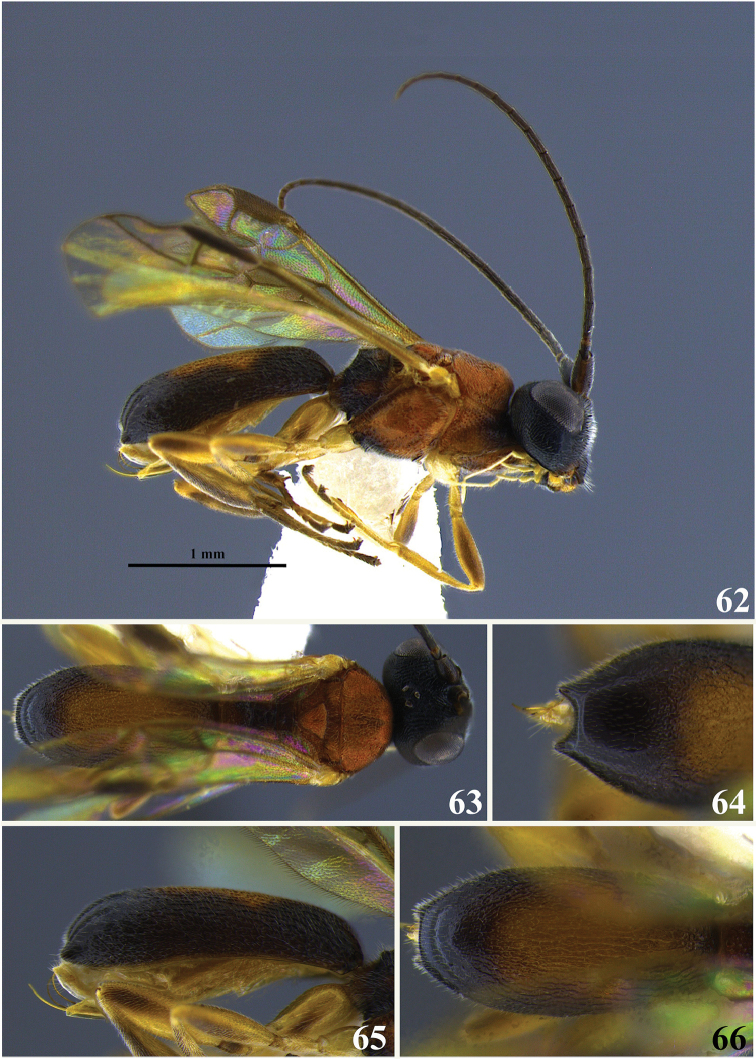
*Leptodrepana ninae*. **62** Female habitus in lateral view **63** female habitus in dorsal view **64** metasoma in dorso-posterior view with a couple of pointing projections **65** metasoma in lateral view **66** metasoma in dorsal view.

#### Holotype female.

BL 2.93 mm; FWL 2.8 mm; CL 1.44 mm; CW 0.4 mm; CL/CW 3.6.

#### Description.

*Color*. Head brownish black, mandibles yellow, blackish brown apically; palpi yellowish white; antennae brown; lateral pronotum mostly orange; mesonotum orange; metanotum brownish black; mesopleuron mostly orange but dark brownish orange posteriorly; propodeum black; fore and middle leg mostly yellowish brown; hind leg with coxa, trochanter and basal portion of femur yellowish white, trochantellus and dorso-apical portions of femur brown, tibia yellowish white ventrally and brown dorsally; wings lightly pigmented with brown venation except parastigma and vein 1M yellow; carapace mostly blackish brown except large oval area of orangish brown.

*Head*. HW 0.825 mm; HL 0.65 mm; HW/HL 1.26; face finely punctuate; genae, vertex and ocellar triangle rugulose-punctate; frons depressed rugulose-punctate with weak median carina; clypeus punctate and apical margin rounded; occipital carina complete; antennae with flagellomeres all longer than wide and decreasing in length apically except penultimate flagellomere almost as long as wide and approximately half the length of ultimate flagellomere.

*Mesosoma*. Pronotum foveolate antero-laterally to rugose at propleural margin; propleuron weakly areolate-rugose; mesoscutum medially areolate-rugose; notauli narrow, visible anteriorly; median and lateral mesonotal lobes punctate; scutellar sulcus with six well-defined depressions, all longer than wide; scutellar disc rugose-weakly punctate; mesopleuron anteriorly rugose, medially shiny and weakly punctate with shallow foveate band at precoxal sulcus, postero-ventrally weakly punctate; propodeum coarsely areolate-rugose with distinct transverse carina raised into small roughly equal medial and lateral flanges.

*Metasoma*. Carapace areolate-rugose basally to shiny and weakly punctate at apex; in dorsal and posterior views, apex terminating in two sharp tubercles deeply arched between; in lateral view, apex terminating in narrow sloping point below midpoint.

**Variation of paratype females.** Median carina distinct; HW 0.83–0.88 mm; HL 0.65–0.75 mm; HW/HL1.1.7–1.27; BL 2.93–3.06 mm; FWL 2.66–2.8 mm; CL 1.44–1.48 mm; CW 0.4–0.44 mm; CL/CW 3.36–3.6.

**Paratype males.** No males.

#### Material examined.

Holotype female: SAN JOSE, Zurqui de Moravia, 1600 m, iii.1991 (P. Hanson) [UWIM]. Paratype data: 1♀, CARTAGO, Dulce Nombre, Vivero Linda Vista, 1400 m, vi–viii.1993; 1♀, PUNTARENAS, Monteverde, San Luis, L-N-250850-449250, 1992 (Z. Fuentes) [INBio, barcode CR1000-805992].

#### Remarks.

*Leptodrepana ninae* is superficially similar to *L. conleyae* but may be distinguished by the following combination of characters. In dorsal and posterior views, the carapace apex terminates in two sharp tubercles that are deeply arched between (Figs 64, 66). The propleural margin of the pronotum is rugose. The mesopleuron is medially shiny and weakly punctate with a shallow foveate band at the precoxal sulcus. The scutellar sulcus has six well-defined depressions. Dorsally the carapace bears a large oval area of orangish brown. In dorsal and posterior views the carapace apex of *L. conleyae* terminates in two small tubercles weakly arched between (Figs 21, 23). The propleural margin of the pronotum is shiny and weakly punctate. The mesopleuron of *L. conleyae* is foveate medially and has a foveate groove at the precoxal sulcus. The scutellar sulcus has five well-defined depressions. The carapace has a baso-medial yellowish white “cape” or patch that extends laterally but not all the way to the lateral margins of the carapace.

#### Etymology.

This species is a patronym in honor of Nina Michelle Zitani for her invaluable aid and advice in all matters entomological.

### Leptodrepana pamelabbas

Taxon classificationAnimaliaHymenopteraBraconidae

Dadelahi & Shaw sp. n.

http://zoobank.org/59FB6236-0D84-4FE8-ABAA-67C9A109351D

Figs 67–71

#### Diagnosis.

In dorsal and posterior views, two small weakly protruding tubercles are visible at the carapace apex. The mesopleuron is medially rugose with wrinkles appearing somewhat parallel. Body mostly brownish black except yellowish brown patches baso-laterally below dorsal carinae; wings lightly pigmented with darker area below stigma covering apical half of 1^st^ submarginal cell and anterior portion of 2^nd^ submarginal cell.

**Figures 67–71.**
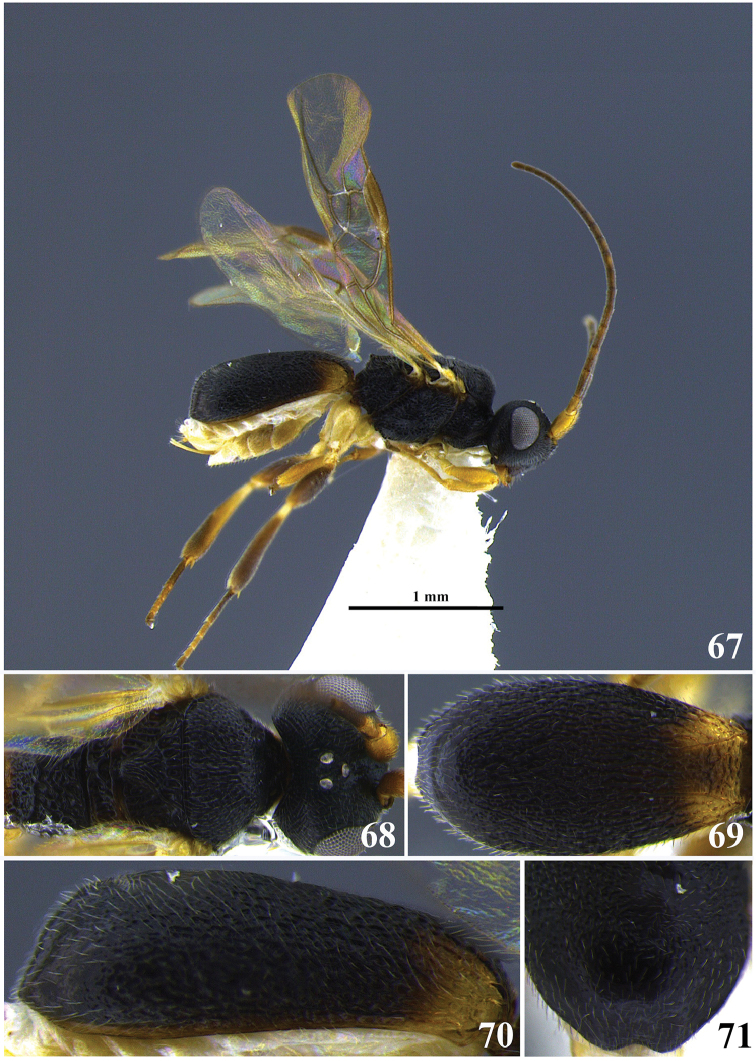
*Leptodrepana pamelabbas*. **67** Female habitus in lateral view **68** female head and mesoscutum in dorsal view **69** metasoma in dorsal view **70** metasoma in lateral view **71** metasoma in dorso-posterior view with a couple of rounded projections.

#### Holotype female.

BL 2.4 mm; FWL 2.3 mm; CL 1.12 mm; CW 0.36 mm; CL/CW 3.11.

#### Description.

*Color*. Head brownish black, clypeus brown, mandibles yellow, blackish brown apically; palpi yellowish white; antennae brown with scape and pedicel yellowish brown; mesosoma brownish black; fore and middle legs with coxa, trochanter and trochantellus yellowish white, femur and tibia yellowish brown; hind leg similar to fore and middle legs but trochantellus, femur and tibia dark brown; tibia with narrow basal band of yellowish white; wings lightly pigmented with darker area below stigma covering apical half of 1^st^ submarginal cell and anterior portion of 2^nd^ submarginal cell; yellowish brown venation; carapace mostly black, with yellowish brown patches baso-laterally below dorsal carinae.

*Head*. HW 0.63 mm; HL 0.48 mm; HW/HL 1.32; face, genae, vertex and ocellar triangle rugulose-punctate; frons depressed impunctate-weakly punctate with median carina faint and weakly foveolate; clypeus weakly punctate and apical margin rounded; occipital carina complete.

*Mesosoma*. Pronotum deeply foveolate antero-laterally to rugose-foveolate at propleural margin; propleuron weakly areolate-rugose; mesoscutum medially with irregular pitted grooves between notauli so that area appears areolate-rugose; notauli indistinct; median and lateral mesonotal lobes rugose-punctate; scutellar sulcus with six well-defined depressions, all longer than wide; scutellar disc punctate; mesopleuron anteriorly rugose and medially regularly rugose (roughly parallel wrinkles); wide foveate band at precoxal sulcus; propodeum coarsely areolate-rugose with distinct transverse carina raised into small and roughly equal medial and lateral flanges.

*Metasoma*. Carapace areolate-rugose to shiny and weakly punctate at apex; in dorsal and posterior views, carapace apex with blunt barely projecting tubercles; in lateral view, apex terminates in broad point.

**Variation of paratype females.** Color: basal quarter of carapace yellowish brown or carapace entirely black/brown; HW 0.63–0.68 mm; HL 0.48–0.53 mm; HW/HL 1.28–1.37; BL 2.4–2.53 mm; FWL 2.3–2.47 mm.; CL 1.12–1.24 mm; CW 0.36–0.44 mm; CL/CW 2.8–3.2.

**Paratype males.** No males.

#### Material examined.

Holotype female: SAN JOSE, Cerro de la Muerte, 26 km N San Isidro, 2100 m, ii–v.1991 (P. Hanson) [UWIM]. Paratype data: 1♀, GUANACASTE, P. N. Santa Rosa, Bosque Humedo mature evergreen dry forest in clearing fully insolated part of the day, 300 m, 28.xii.1985–18.i.1986 (I. Gauld & D. Janzen).

#### Remarks.

*Leptodrepana pamelabbas* and *L. alexisae* are similar in size, shape, and color. However, the following characters may be used to separate the two species. In dorsal and posterior views, two small weakly protruding tubercles are visible at the carapace apex of *L. pamelabbas* (Figs 69, 71). The mesopleuron is medially rugose with wrinkles appearing somewhat parallel. At the precoxal sulcus there is a wide foveate groove. In females, the flagellum is uniform in width. In dorsal view, the carapace apex of *L. alexisae* lacks projecting tubercles instead it has a protruding flange (Fig. 6). In posterior view, two planar points, either strongly or weakly arched between, are visible (Fig. 4). The mesopleuron has a small shiny impunctate area medially with the immediate surrounding area foveolate. The precoxal sulcus appears foveolate. In females, the flagellum is slightly dilated medially.

#### Etymology.

This species name is an arbitrary arrangement of letters to form a euphonious combination.

### Leptodrepana ronnae

Taxon classificationAnimaliaHymenopteraBraconidae

Dadelahi & Shaw sp. n.

http://zoobank.org/F327E81D-9B3C-42C9-B599-60D52CCEEC46

Figs 72–76

#### Diagnosis.

In dorsal view, apex appears rounded or with barely protruding truncated flange below rounded dorsum of carapace; in lateral view, carapace apex terminates in narrow sloping point below midline. Presence of a transverse carina at the carapace apex in posterior view. Scutellar sulcus with three well-defined depressions, all longer than wide. Body mostly brownish black except flagellum brown that bears a yellowish white annulus at flagellomeres 4–6, and carapace with basal half yellowish white.

**Figures 72–76.**
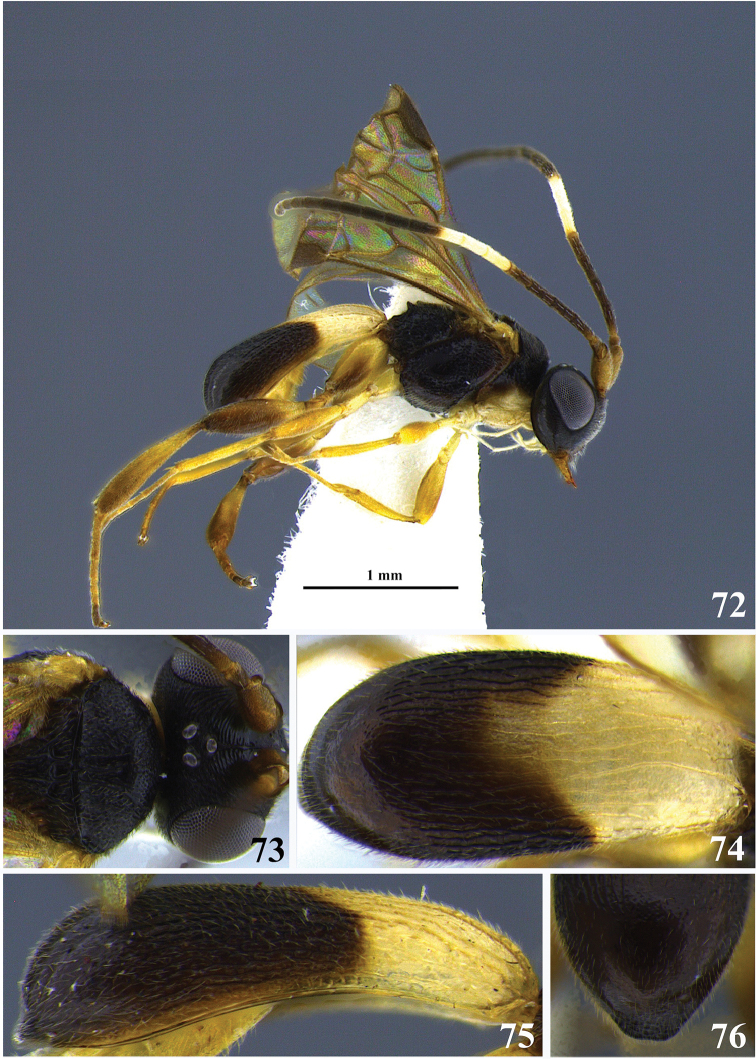
*Leptodrepana ronnae*. **72** Female habitus in lateral view **73** female head and mesoscutum in dorsal view **74** metasoma in dorsal view **75** metasoma in lateral view **76** metasoma in dorso-posterior view terminating truncated.

#### Holotype female.

BL 2.87 mm; FWL 2.6 mm; CL 1.32 mm; CW 0.4 mm; CL/CW 3.3.

#### Description.

*Color*. Head brownish black, mandibles yellow, blackish brown apically; palpi yellowish white; antennae brown, except for flagellomeres 4–6 yellowish white, scape and pedicel yellowish brown; mesosoma mostly black, pronotum yellowish white postero-laterally, propleuron yellowish white; fore and middle legs with coxa and trochanter yellowish white, trochantellus, femur and tibia yellowish brown; hind leg similar except brown and coxa with apical brown patch; wings lightly pigmented with darker area below stigma covering apical half of 1^st^ submarginal cell and anterior portion of 2^nd^ submarginal cell; carapace blackish brown with basal half yellowish white.

*Head*. HW 0.85 mm; HL 0.7 mm; HW/HL 1.21; face, genae, vertex and ocellar triangle rugulose-punctate; frons depressed punctate with distinct parallel lineation lateral to median carina; clypeus punctate and apical margin rounded; occipital carina complete; antennae with flagellum slightly dilated medially.

*Mesosoma*. Pronotum mostly shiny and impunctate but rugose-punctate near tegula; propleuron weakly areolate-rugose; mesoscutum medially with shallow irregular pitted grooves between notauli, mesonotal lobes rugose-punctate; notauli visible anteriorly; scutellar sulcus with three well-defined depressions, all longer than wide; scutellar disc rugose-punctate; mesopleuron anteriorly rugose, medially shiny and weakly punctate, some shallow irregularly shaped pits at precoxal sulcus, and weakly punctate to impunctate postero-ventrally; propodeum coarsely areolate-rugose with distinct transverse carina raised into medial and lateral flanges, lateral flanges greatly protruding approximately 3× the size of median flanges.

*Metasoma*. Carapace areolate-rugose to punctate at apex; in dorsal view, apex appears rounded or with barely protruding truncated flange below rounded dorsum of carapace; in lateral view, carapace apex terminates in narrow sloping point below midline; in posterior view, apex terminates in shallowly arched ridge or carina.

**Variation of paratype females.** No paratype females.

**Paratype males.** No males.

#### Material examined.

Holotype female: PUNTARENAS, San Vito, Jardin Bot. Las Cruces, 1200 m, vi–vii.1988 (P. Hanson) [UWIM].

#### Remarks.

*Leptodrepana ronnae* is similar to *L. strategeri* in shape and color. It may be easily distinguished from *L. strategeri* by the presence of a transverse carina at the carapace apex in posterior view. Also when viewed posteriorly, the carapace apex appears distinctly wrinkled or rugulose. The scutellar sulcus has only three well-defined depressions and the mesopleuron has shallow irregularly shaped pits at the precoxal sulcus. In females, the flagellum is brown but bears a yellowish white annulus at flagellomeres 4–6. *Leptodrepana strategeri* lacks a transverse carina at the carapace apex in posterior view. The carapace is shiny and weakly punctate at the apex. The scutellar sulcus has 4–5 well-defined depressions and the mesopleuron has a scrobiculate groove at the precoxal sulcus. In females the flagellum is light brown.

#### Etymology.

This species name is a patronym for Ronnae Lynn Nemitz, a close friend of SDD, always ready to point out that we do not know everything.

### Leptodrepana rosanadana

Taxon classificationAnimaliaHymenopteraBraconidae

Dadelahi & Shaw sp. n.

http://zoobank.org/16AF8298-02ED-4B07-8BE7-3163EC69EC15

Figs 77–81 , 127

#### Diagnosis.

In lateral and dorsal views, the carapace apex is rounded; in posterior view, the carapace apex is rounded with the posterior margin weakly arched. The scutellar disc is distinctly bulging and punctate. Body mostly black except basal third of carapace with median yellowish white oval shape; the wings are suffused with dark brown pigment except for an obfuscate circular area in the basal cell, directly below the stigma in the first submarginal cell, and a linear area parallel to the vein 1A in subbasal cell.

**Figures 77–81.**
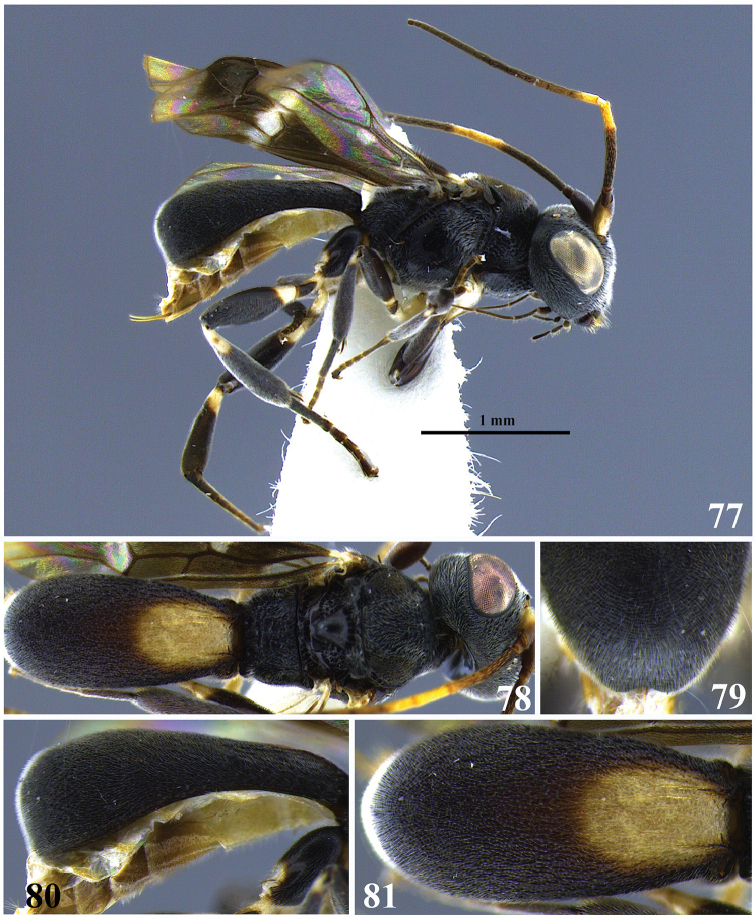
*Leptodrepana rosanadana*. **77** Female habitus in lateral view **78** female habitus in dorsal view **79** metasoma in dorso-posterior view with a small, median, ventral depression **80** metasoma in lateral view **81** metasoma in dorsal view.

#### Holotype female.

BL 3.27 mm; FWL 2.3 mm; CL 1.44 mm; CW 0.44 mm; CL/CW 3.27.

#### Description.

*Color*. Head black, mandibles yellow, blackish brown apically; palpi dark brown; antennae dark brown except flagellomeres 2–4 yellowish white; mesosoma black; middle and fore leg coxae and dorsum of trochanter yellowish white; femur and tibia dark brown both with narrow basal bands of yellowish white; hind leg same as fore and middle legs except coxa basal and apical portions of coxa and trochanter yellowish white; wings suffused with dark brown pigment except for obfuscate circular area in basal cell, directly below stigma in first submarginal cell, and linear area parallel to vein 1A in subbasal cell; venation brown; basal third of carapace with median yellowish white oval shape, remainder of carapace black.

*Head*. HW 1.03 mm; HL 0.85 mm; HW/HL 1.21; face, genae, vertex and ocellar triangle coarsely rugulose-punctate; frons depressed and pubescent, pubescence obscuring sculpture and posterior portion of median carina; clypeus coarsely punctate and apical margin rounded; occipital carina complete.

*Mesosoma*. Pronotum laterally deeply foveate; propleuron weakly areolate-rugose; mesoscutum coarsely punctate; notauli narrow and visible anteriorly; scutellar sulcus with six well-defined depressions, all longer than wide; scutellar disc distinctly bulging and punctate; anterior scutellar depression foveate; mesopleuron anteriorly rugose, medially shiny and impunctate, foveate at precoxal sulcus, foveolate to weakly punctate postero-ventrally; propodeum coarsely areolate-rugose with distinct transverse carina raised into medial and lateral flanges, median flanges reduced and lateral flanges at least twice size of median flanges.

*Metasoma*. Carapace basally areolate-rugose to rugulose at apex; in dorsal and lateral views, apex of carapace rounded; in posterior view, carapace apex rounded with posterior margin weakly arched.

**Variation of paratype females.** HW 1.13 mm; HL 0.875 mm; HW/HL 1.29; BL 3.5 mm; FWL 2.6 mm; CL 1.6 mm; CW 0.56 mm; CL/CW 2.86.

**Variation of paratype males.** Similar to females except posterior margin of carapace apex rounded not weakly arched; antennae broken; HW 1.11 mm; HL 0.86 mm; HW/HL 1.29; BL 3.54 mm; FWL 2.8 mm; CL 1.49 mm; CW 0.44 mm; CL/CW 3.38.

#### Material examined.

Holotype female: PUNTARENAS, R. F. Golfo Dulce, 3 km SW Rincon, primary forest, 10 m, iii.1993 (P. Hanson) [UWIM]. Paratype data: 1♀, same data as holotype; 1♂, same province except P. N. Corcovado, Est. Sirena, 0–100 m, L-S-270500-508300, vi.1991 (G. Fonseca) [INBio, barcode CR1000-669411].

#### Remarks.

*Leptodrepana rosanadana* has unique characters that easily distinguish it from other Costa Rican species. In lateral and dorsal views, the carapace apex is rounded (Figs 80, 81). In posterior view, the carapace apex is rounded with the posterior margin weakly arched (Fig. 79). The carapace apex is rugulose. The scutellar disc is distinctly bulging and punctate. The anterior scutellar depression is foveate. The mesopleuron is medially shiny and impunctate and foveate at the precoxal sulcus. The medial flanges of the propodeal carina are reduced so that the lateral flanges appear at least twice the size of the median flanges. The wings are suffused with dark brown pigment except for an obfuscate circular area in the basal cell, directly below the stigma in the first submarginal cell, and a linear area parallel to the vein 1A in subbasal cell (Fig. 127).

*Leptodrepana rosanadana* showed *Ascogaster*-like variation in the scutellar disc. In *Leptodrepana* the disc tends to be characteristically flattened and polished while, in *Ascogaster*, it tends to be bulging or convex and sculptured. *Leptodrepana rosanadana* had a noticeably bulging coarsely sculptured scutellar disc. However, we firmly believe this species to be within the genus *Leptodrepana*. The antennae consist of 17 flagellomeres of uniform width, the ventral cavity is roughly the same length as the carapace, and the ocellar triangle is equilateral in shape.

#### Etymology.

This species name is an arbitrary arrangement of letters to form a euphonious combination.

### Leptodrepana schuttei

Taxon classificationAnimaliaHymenopteraBraconidae

Dadelahi & Shaw sp. n.

http://zoobank.org/2D77F1B5-14CF-4F51-B448-F06FDC4DE5EB

Figs 82–86

#### Diagnosis.

Carapace apex terminates in single small point; in lateral view apex terminates in broad point at midline. Head brownish black, mesosoma mostly orange, wings suffused with light yellowish brown pigment, darker area below stigma, basal third of carapace yellowish white and apical 2/3 of carapace blackish brown.

**Figures 82–86.**
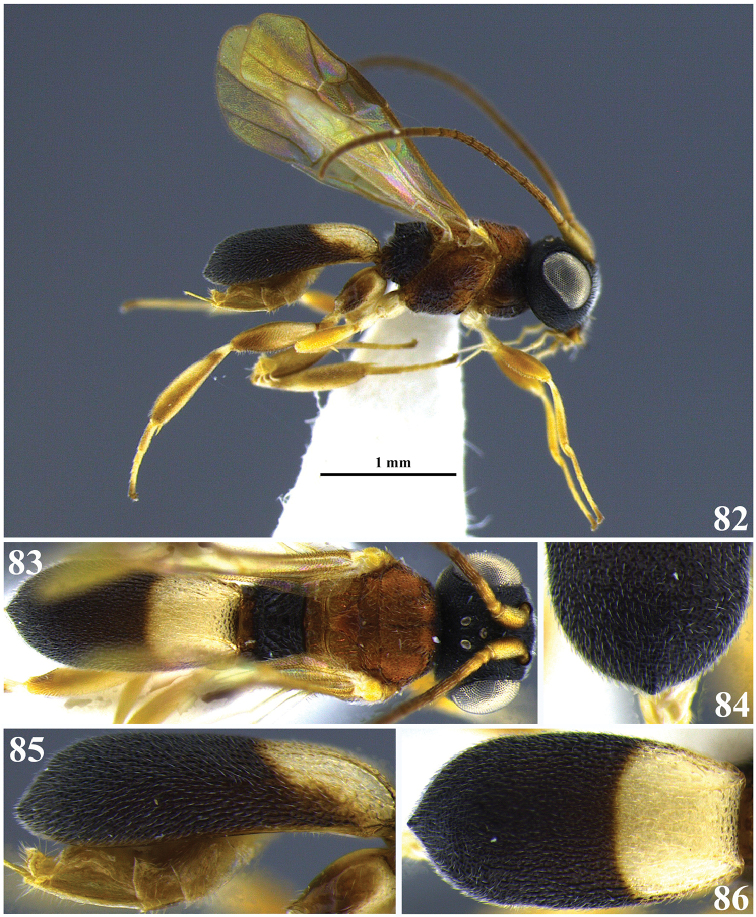
*Leptodrepana schuttei*. **82** Female habitus in lateral view **83** female habitus in dorsal view **84** metasoma in dorso-posterior view with a pointing projection **85** metasoma in lateral view **86** metasoma in dorsal view.

#### Holotype female.

BL 3.07 mm; FWL 2.4 mm; CL 1.32 mm; CW 0.36 mm; CL/CW 3.6.

#### Description.

*Color*. Head brownish black, mandibles yellow, blackish brown apically; apical margin of clypeus orange; palpi yellowish white; antennae brown with scape and pedicel and first flagellomere (basal) yellowish white; mesosoma mostly orange except posterior half of mesopleuron black, metapleuron, and propodeum black; middle and fore legs yellowish white; hind leg mostly yellowish white except apical portion of coxa brown; wings suffused with light yellowish brown pigment, darker area below stigma covering apical half of 1^st^ submarginal cell and anterior portion of 2^nd^ submarginal cell; venation yellowish brown; basal third of carapace yellowish white and apical 2/3 of carapace blackish brown.

*Head*. HW 0.9 mm; HL 0.75 mm; HW/HL 1.2; face, genae, vertex and ocellar triangle rugulose-punctate; frons depressed, weakly punctate with fine parallel lineation lateral to weak median carina; clypeus punctate and apical margin rounded; occipital carina complete.

*Mesosoma*. Pronotum foveate antero-laterally to foveolate-rugose at propleural margin; propleuron weakly areolate-rugose; mesoscutum medially with irregular parallel pitted grooves between notauli difficult to distinguish and appears areolate-rugose; notauli distinct and visible anteriorly; median and lateral mesonotal lobes rugose-punctate; scutellar sulcus with 5 well-defined depressions, all longer than wide; scutellar disc punctate; mesopleuron anteriorly rugose, medially deeply foveate, wide scrobiculate groove at precoxal sulcus, foveate postero-ventrally; propodeum coarsely areolate-rugose with distinct transverse carina raised into small and roughly equal medial and lateral flanges.

*Metasoma*. Carapace completely areolate-rugose; in dorsal and posterior views, carapace apex terminates in single small point; in lateral view apex terminates in broad point at midline.

**Variation of paratype females.** HW 0.85–0.95 mm; HL 0.725–0.8 mm; HW/HL 1.17–1.2; BL 2.6–3.2 mm; FWL 2.7–2.47 mm; CL 1.24–1.4 mm; CW 0.36–0.4 mm; CL/CW 3.1–3.8.

**Paratype males.** No males.

#### Material examined.

Holotype female: GUANACASTE, P. N. Santa Rosa, site #12, Bosque Humedo mature evergreen dry forest more or less fully shaded as possible, 300 m, 27.ix–18.x.1986 (I. Gauld & D. Janzen) [UWIM]. Paratype data: 1♀, same data as holotype; 3♀, same data except site #5, Bosque San Emilio, 50 year old deciduous forest in clearing fully insolated part of the day, 6–27.ix.1986; 1♀, same data except more or less fully shaded as possible, 18.x–8.xi.1986.

#### Remarks.

*Leptodrepana schuttei* may be confused with *L. atalanta*. They are similar in color and the carapace apex of both species terminates in a single point. *Leptodrepana schuttei* may be distinguished from this species by the following combination of characters. The notauli of *L. schuttei* is narrow and a scrobiculate groove is present at the precoxal sulcus. The posterior mesopleuron and the propodeum are black. The basal third of the carapace is completely yellowish white. The notauli of *L. atalanta* are wide and shallow. There is a wide band at the precoxal sulcus formed by at least two shallow foveate grooves. The mesopleuron and propodeum are entirely orange. The carapace has a baso-median patch of yellowish white that does not extend to the lateral margins of the carapace.

#### Etymology.

This species is a patronym for Vincent Paul Schutte. After all the science fiction watched together, it seemed fitting that something as “alien” as a koinobiont parasitoid should bear his name.

### Leptodrepana scottshawi

Taxon classificationAnimaliaHymenopteraBraconidae

Dadelahi sp. n.

http://zoobank.org/251BEE78-791A-41F9-A051-F1E7AC11B9D9

Figs 87–91

#### Diagnosis.

The carapace apex appears coarsely rugulose-punctate and terminates in two protruding points or tubercles that are visible in both dorsal and posterior views. Medially, the mesoscutum is divided into 5–6 neat parallel-pitted grooves and scutellar disc is very flat. Mesosoma orange; head and metasoma mostly blackish brown except with yellowish white patches baso-laterally below dorsal carinae.

**Figures 87–91.**
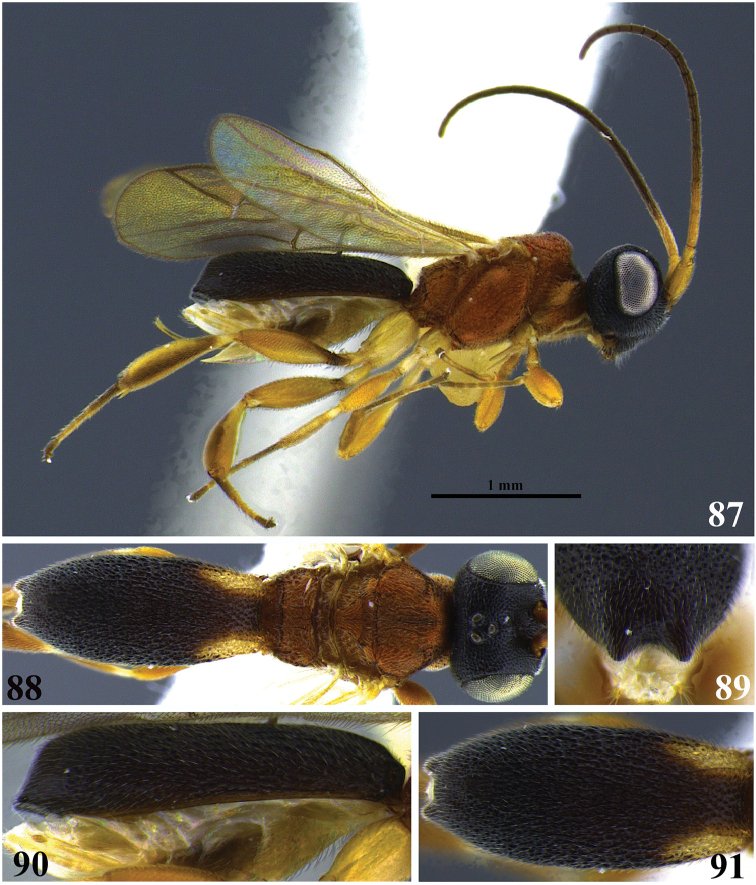
*Leptodrepana scottshawi*. **87** Female habitus in lateral view **88** female habitus in dorsal view **89** metasoma in dorso-posterior view with a couple of pointing projections **90** metasoma in lateral view **91** metasoma in dorsal view.

#### Holotype female.

BL 2.9 mm; FWL 2.26 mm; CL 1.44 mm; CW 0.36 mm; CL/CW 4.

#### Description.

*Color*. Head blackish brown, mandibles mostly yellowish brown but blackish brown basally and apically; palpi yellowish white; antennae dark brown with scape, pedicel and first two basal flagellomeres yellowish white; mesosoma orange; legs orange except middle coxae and trochanter yellowish white, hind trochanter yellowish white and tibia of all legs with basal yellowish white band; venation brown with parastigma and vein 1M lighter in color, yellow-brown; carapace brownish black with yellowish white patches baso-laterally below dorsal carinae.

*Head*. HW 0.88 mm; HL 0.68 mm; HW/HL 1.3; face, vertex genae and ocellar triangle rugulose-punctate; frons depressed, rugulose-punctate with fine parallel lineation lateral to median carina; clypeus punctate and apical margin rounded; occipital carina complete.

*Mesosoma*. Pronotum foveate antero-laterally to weakly rugose at propleural margin; propleuron weakly areolate-rugose; mesoscutum medially with 5–6 distinct regular parallel lacunose grooves between notauli; notauli narrow and visible anteriorly; median and lateral mesonotal lobes rugulose-punctate; scutellar sulcus with 6–7 well-defined depressions, all longer than wide; scutellar disc punctate and very flat; mesopleuron anteriorly rugose, medially rugose-foveate and, at precoxal sulcus, two foveate grooves form an wide band; propodeum coarsely areolate-rugose with distinct transverse carina raised into small and roughly equal medial and lateral flanges.

*Metasoma*. Carapace areolate-rugose to coarsely rugulose-punctate at apex; in posterior view, apex terminating in two points shallowly arched between; in dorsal view, apex terminates in two points visible below rounded carapace dorsum; in lateral view, apex terminates in sloping point below midline; ventral cavity almost reaching apex.

**Variation of paratype females.** Color: middle and hind tibia brown with basal yellowish white band; carapace entirely black or with baso-lateral white patches continuing dorsally sometimes meeting dorsally; frons weakly punctate with lineation lateral to median carina weak or well-defined; pronotum smooth at propleural margin; HW 0.73–0.88 mm; HL 0.58–0.68 mm; HW/HL 1.23–1.3; BL 2.5–3.0 mm; FWL 1.8–2.4 mm; CL 1.6–1.44 mm; CW 0.36–0.4 mm; CL/CW3.2–3.89.

**Variation of paratype males.** Similar to females except all legs with coxa, trochanter, and trochantellus yellowish white; mesopleuron medially weakly punctate and foveate at precoxal sulcus but not forming a wide grooved band; HW 0.73–0.8 mm; HL 0.58–0.63 mm; HW/HL 1.26–1.28; BL 2–2.27 mm; FWL 1.87–2.13 mm; CL 1.16–1.4 mm; CW 0.36 mm; CL/CW 3.2–3.89.

#### Material examined.

Holotype female: PUNTARENAS, Golfo Dulce 24 km W Piedras Blancas, 200 m, vi–viii.1989 (P. Hanson) [UWIM]. Paratype females: 1♀, HEREDIA, 3 km S Puerto Viejo, OTS, La Selva, 100 m, xi.1992; 1♀, GUANACASTE, Est. Mengo, SW Volcan Cacao, 1100 m, 1988–1989 (No collector listed); 2♀, same data except Est. Pitilla, 9 km S Santa Cecilia, 700 m, v.1989 (I. Gauld); 1♀, 1♂, LIMON, R. B. Hitoy Cerere, Est. Hitoy Cerere, 100 m, 19–29.iv.1992 (E. Lopez) L-N 184200,643300 [INBio]; 1♀, Sector Cerro Cocori, Fca. De E. Rojas, 150 m, L-N-286000,567500, 5.vi–5.vii.1992 (No Collector listed) [INBio]; 1♀, 3♂, Las Cruces biological station, Pacific slope, 1100 m, light trap, 6–9.vii.1999 (N. Zitani); 3♀, same as holotype; remaining paratypes are from PUNTARENAS; 1♀, R. F. Golfo Dulce, 3 km SW Rincon 10 m, ii–iii.1989 (Hanson & Gauld); 2♀, same data except x–xii.1990; 2♀, same data except vi.1991; 1♀, same data except ii.1993; 5♀, road to Rincon, 10 km W of Pan-American Hwy, 100 m, iii–v.1989 (Hanson & Gauld); 1♀, Corcovado, Est Sirena, 50 m, x–xii.1990; 1♀, same data except 0–100 m, L-S-270500,508800, xii.1991 (G. Fonseca) [INBio]; 1♀, 3♂, P. N. Manuel Antonio, Quepos, 80 m, L-S-370900,448800 #1181 iv.1992 (C.Cano) [INBio]; 2♀, R. B. Carara, Est. Queb. Bonito, 50 m, L-N-194500, 469850, ii.1993 (R. Guzman) [INBio].

#### Remarks.

*Leptodrepana scottshawi* is easily distinguished from other Costa Rican *Leptodrepana*. Medially, the mesoscutum is divided into 5–6 neat parallel pitted grooves and scutellar disc is very flat. The carapace apex appears coarsely rugulose-punctate and terminates in two protruding points or tubercles that are visible in both dorsal and posterior views.

#### Etymology.

This species name by SDD is a patronym for her advisor Scott Shaw, in appreciation for all the encouragement, advice, and occasional lash of the whip he has been obliged to give over the years.

### Leptodrepana shriekae

Taxon classificationAnimaliaHymenopteraBraconidae

Dadelahi & Shaw sp. n.

http://zoobank.org/52E77A3A-2FC8-455E-91A6-3DC66E2EDC15

Figs 92–96

#### Diagnosis.

Body small, less than 2.5 mm; the carapace apex has two protruding points or tubercles that are visible in both dorsal and posterior view; the apex is shiny and weakly punctate; the mesopleuron is medially shiny-impunctate and it is deeply foveate at the precoxal sulcus. Body mottled brownish orange in color with a very characteristic median orange square patch on mesoscutum.

**Figures 92–96.**
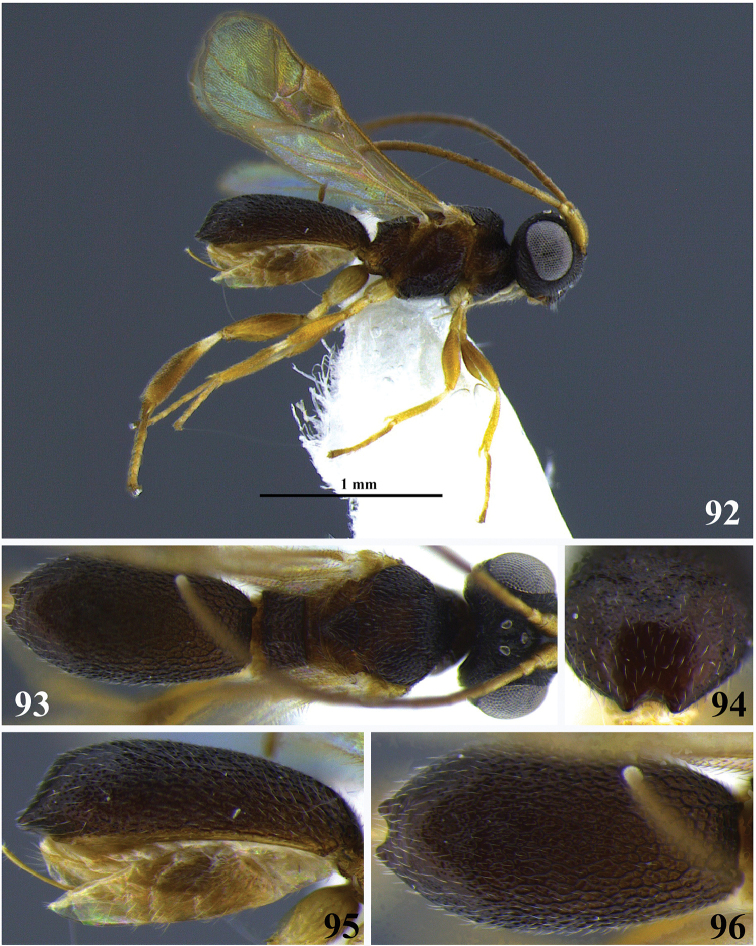
*Leptodrepana shriekae*. **92** Female habitus in lateral view **93** female habitus in dorsal view **94** metasoma in dorso-posterior view with a couple of pointing projections **95** metasoma in lateral view **96** metasoma in dorsal view.

#### Holotype female.

BL 2.13 mm; FWL 1.67 mm; CL 0.96 mm; CW 0.32 mm; CL/CW 3.

#### Description.

*Color*. Head blackish brown, mandibles mostly yellowish brown but blackish brown basally and apically; palpi yellowish white; antennae dark brown with scape, pedicel and first flagellomere (basal) yellowish white; mesosoma mostly brownish black except mesoscutum with median yellowish brown square patch and anterior scutellar depression surrounding scutellar disc yellowish brown; legs yellow with faint basal white band on tibia; wings lightly pigmented with darker area below stigma covering apical half of 1^st^ submarginal cell and anterior portion of 2^nd^ submarginal cell; yellowish brown venation; carapace brownish black.

*Head*. HW 0.5 mm; HL 0.53 mm; HW/HL 0.94; face, vertex and ocellar triangle rugulose-punctate, genae weakly so; frons depressed, weakly punctate, and median carina lacking; clypeus punctate and apical margin rounded; occipital carina complete.

*Mesosoma*. Pronotum foveate antero-laterally to weakly rugose at propleural margin; propleuron weakly areolate-rugose; mesoscutum medially with irregular parallel pitted grooves between notauli difficult to distinguish and appears areolate-rugose; notauli narrow and visible anteriorly; median and lateral lobes rugulose-punctate; scutellar sulcus with six well-defined depressions, all longer than wide; scutellar disc punctate; mesopleuron anteriorly rugose, medially impunctate and shiny deeply foveate at precoxal sulcus and impunctate to weakly punctate postero-ventrally; propodeum coarsely areolate-rugose with distinct transverse carina raised into small and roughly equal medial and lateral flanges.

*Metasoma*. Carapace areolate-rugose basally graduating to shiny and weakly punctate at apex; but weak; in posterior view, apex terminating in two points shallowly arched between; in dorsal view, apex terminates in two points visible below rounded carapace dorsum; in lateral view, apex terminates in sloping point below midline; ventral cavity almost reaching apex.

**Variation of paratype females.** Color: clypeus orange; color pattern more pronounced with yellowish cream tibial band and orange square patch on mesoscutum in stark contrast with surrounding areas; median carina weak medially; points at apex of carapace deeply arched between; HW 0.58–0.7 mm; HL 0.48–0.56 mm; HW/HL 1.2–1.3; BL 1.9–2.3 mm; FWL 1.5–1.9 mm; CL 0.9–1.1 mm; CW 0.32 mm; CL/CW 2.8–3.4.

**Paratype males.** Similar to females except clypeus yellow/orange; antennae broken in male examined; flagellum yellowish white; face sometimes with orange markings below antennae; mesoscutum without median orange square patch coarsely punctate medially; anterior scutellar depression surrounding scutellar disc same color as disc; ventral cavity distal carapace apex by at least twice the distance in females; HW 0.58 mm; HL 0.5 mm; HW/HL 1.2; BL 2.1 mm; FWL broken; CL 0.92 mm; CW 0.36 mm; CL/CW 2.56.

#### Material examined.

Holotype female: SAN JOSE, San Antonio de Escazu, 1300 m, i–ii.1989 (W. Eberhard) [UWIM]. Paratype females: 3♀, GUANACASTE, P. N. Santa Rosa, Bosque Humedo mature evergreen dry forest in clearing fully isolated part of the day, 300 m, 28.xii.1985–18.i.1986 (I. Gauld & D. Janzen); 8♀, 1♂, same data except 29.xi–20.xii.1986.

#### Remarks.

*Leptodrepana shriekae* may be distinguished from other Costa Rican species by the following combination of characters: the body is small, less than 2.5 mm; the carapace apex has two protruding points or tubercles that are visible in both dorsal and posterior view; the apex is shiny and weakly punctate; the mesopleuron is medially shiny-impunctate and it is deeply foveate at the precoxal sulcus. *Leptodrepana shriekae* is an interesting mottled brownish orange in color with a very characteristic median orange square patch on mesoscutum.

#### Etymology.

This species name is a patronym for Erika L. Smith, affectionately known as Shriek since the age of fourteen, in appreciation for all the commiseration she has provided to SDD over the years.

### Leptodrepana sohailae

Taxon classificationAnimaliaHymenopteraBraconidae

Dadelahi & Shaw sp. n.

http://zoobank.org/17F2BFC9-DE52-4B94-9CE1-874D3348358B

Figs 97–101

#### Diagnosis.

Body small, less than 2.5 mm in length. The carapace apex terminates in a single point, which is visible in both dorsal and posterior views. The carapace apex is shiny-impunctate. The mesopleuron is medially shiny and weakly punctate and the precoxal sulcus is defined by a scrobiculate groove. The body is dark brown in color.

**Figures 97–101.**
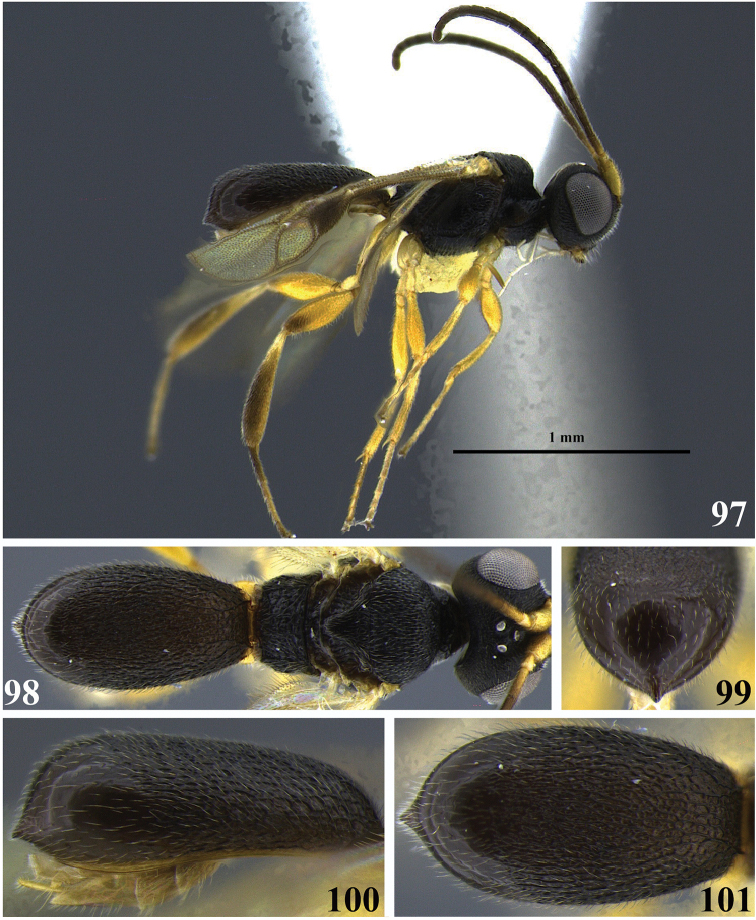
*Leptodrepana sohailae*. **97** Female habitus in lateral view **98** female habitus in dorsal view **99** metasoma in dorso-posterior view with a pointing projection **100** metasoma in lateral view **101** metasoma in dorsal view.

#### Holotype female.

BL 1.7 mm; FWL 1.4 mm; CL 0.8 mm; CW 0.2 mm; CL/CW 4.

#### Description.

*Color*. Head brown, mandibles mostly yellowish brown but blackish brown basally and apically; palpi yellowish white; antennae dark brown, darkest apically with scape, pedicel and first two basal flagellomeres yellowish brown; mesosoma brown; legs yellow with dark area dorso-apically on femur and tibia with faint yellowish white band basally; venation yellowish brown; carapace brown.

*Head*. HW 0.58 mm; HL 0.43 mm; HW/HL 1.35; face, vertex and ocellar triangle rugulose-punctate, genae weakly so; frons depressed, weakly punctate and median carina weak; clypeus punctate and apical margin rounded; occipital carina complete.

*Mesosoma*. Pronotum weakly foveolate-punctate antero-laterally to smooth at propleural margin; propleuron weakly areolate-rugose; mesoscutum medially with irregular parallel pitted grooves between notauli difficult to distinguish and appears areolate-rugose; notauli narrow and visible anteriorly; median and lateral mesonotal lobes rugose-foveolate; scutellar sulcus with 5–6 well-defined depressions, all longer than wide; scutellar disc weakly punctate; mesopleuron anteriorly rugose, medially suture shiny and weakly punctate, scrobiculate groove at precoxal sulcus; propodeum coarsely areolate-rugose with distinct transverse carina raised into small and roughly equal medial and lateral flanges.

*Metasoma*. Carapace areolate-rugose to shiny and impunctate at apex; in dorsal view, apex terminates in single point; in lateral view, apex terminates in sloping point approximately at midline; ventral cavity almost reaching apex.

**Variation of paratype females.** Color: color patterns ,tibial basal band and basal portions of antennae, well differentiated; tibia of hind leg dark brown dorsally ; HW 0.58–0.65 mm; HL 0.43–0.48 mm; HW/HL 1.35–1.4; BL 1.7–2.2 mm; FWL 1.4–2.1 mm; CL 0.8–1.0 mm; CW 0.2–0.36 mm; CL/CW2.8–4.

**Paratype males.** No males.

#### Material examined.

Holotype female: ALAJUELA, San Pedro de la Tigra Cacao, 200 m, iii–iv.1990 (R. Cespedes) [UWIM]. Paratype female: 1♀, GUANACASTE, Est. Pitilla 9 km S Santa Cecilia, 700 m, v.1989 (I. Gauld).

#### Remarks.

*Leptodrepana sohailae* may be distinguished from other Costa Rican species by the following combination of characters. The body is small, being less than 2.5 mm in length. The carapace apex terminates in a single point, which is visible in both dorsal and posterior views. The carapace apex is shiny-impunctate. The mesopleuron is medially shiny and weakly punctate and the precoxal sulcus is defined by a scrobiculate groove. The body is dark brown in color.

#### Etymology.

This species name is a patronym named in honor of a sister of SDD, Sohaila Catherine Dadelahi.

### Leptodrepana sorayae

Taxon classificationAnimaliaHymenopteraBraconidae

Dadelahi & Shaw sp. n.

http://zoobank.org/7B0661BF-5C7D-4C17-94F2-28750691EA53

Figs 102–106 , 128

#### Diagnosis.

In lateral view, the carapace bears a rounded flange posterior to the dorso-apical point. In posterior view the carapace apex terminates in a single broad point below which is a strongly arched medially notched flange. Body mostly yellowish orange; wings suffused with smoky brown pigmentation except for a white band running from the and including the parastigma.

**Figures 102–106.**
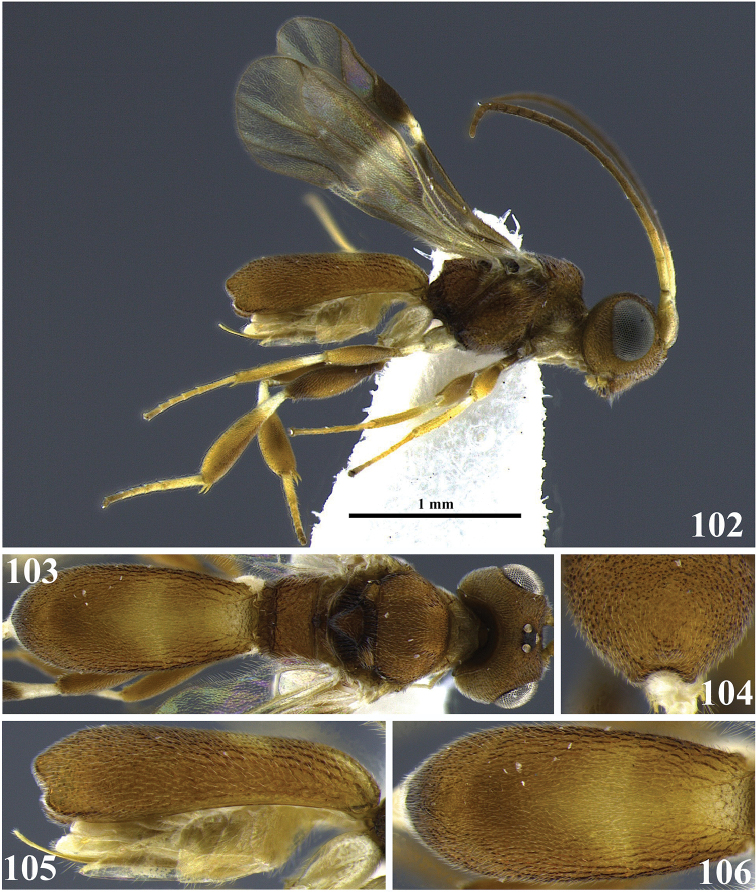
*Leptodrepana sorayae*. **102** Female habitus in lateral view **103** female habitus in dorsal view **104** metasoma in dorso-posterior view with a couple of terminal projections **105** metasoma in lateral view **106** metasoma in dorsal view.

#### Holotype female.

BL 2.6 mm; FWL 2.13 mm; CL 1.2 mm; CW 0.4 mm; CL/CW 3.3.

#### Description.

*Color*. Head yellow, mandibles yellow, blackish brown apically; palpi yellowish white; antennae brown with scape, pedicel and first two flagellomeres (basal) yellow; mesosoma yellowish orange except scutellar sulcus dark brown, propleural margin of pronotum and propleuron yellowish white; fore leg coxa brown, trochanter brown and apically white, trochantellus yellow, femur, tibia and tarsus orangish yellow; middle leg coxa white, trochanter mostly white, trochantellus mostly brown, femur, tibia and tarsus orangish yellow; hind leg coxa white, trochanter mostly white, trochantellus mostly brown, femur orangish yellow but dark brown dorsally, tibia white basally and orangish brown apically, tarsus orangish brown; wings suffused with smoky brown pigmentation except for white band running from parastigma (white) over veins RS+Ma, RS+Mb, 1m-cu, 2Cua and basal most portions of veins 2RS, 2M, and 3CU; remaining venation dark brown; carapace mostly orange, basally with median yellowish white patch between dorsal carinae, posterior margin of basal third of carapace with very faint ridge followed by transverse crescent shaped yellowish white patch.

*Head*. HW 0.9 mm; HL 0.75 mm; HW/HL 1.2; face and genae rugulose punctate; vertex and ocellar triangle coarsely rugulose-punctate; frons depressed, coarsely punctate with fine parallel lineation transverse to median carinae; clypeus punctate and apical margin rounded; occipital carina complete.

*Mesosoma*. Pronotum foveate antero-laterally to rugose-foveate at propleural margin; propleuron weakly foveolate-rugose; mesoscutum medially with 3–4 irregular parallel pitted grooves between notauli so that area appears neatly divided parallel lines; notauli narrow and visible anteriorly; medial mesonotal lobe granulate, lateral mesonotal lobes weakly areolate-rugose; scutellar sulcus with 6–7 well-defined depressions, all longer than wide; scutellar disc punctate; mesopleuron anteriorly rugose, medially foveate, scrobiculate groove at precoxal sulcus followed by a foveate groove together appearing as a wide grooved depression; propodeum coarsely areolate-rugose with distinct transverse carina raised into small, roughly equal, medial and lateral flanges.

*Metasoma*. Carapace areolate-rugose to weakly areolate-rugose at apex; in posterior view apex terminating in a single broad point with arched flanges beneath; in dorsal view, apex terminates in single point; in lateral view, posterior to apical point is rounded flange; ventral cavity distal apex.

**Variation of paratype females.** Color: head yellow to orange; pronotum orange to yellowish white at propleural margin; propleuron orange to yellowish white; mesosoma orange/brown; anterior and posterior portion of mesopleuron brown/black; carapace orange/brown; pattern on carapace faint; propleural margin of pronotum rugose; propleuron weakly areolate-rugose; HW 0.8–0.9 mm; HL 0.7–0.73 mm; HW/HL 1.1–1.2; BL 2.6–3.1 mm; FWL 2.1–2.3 mm; CL 1.2–1.5 mm; CW 0.36–0.44 mm; CL/CW 3.2–3.7.

**Variation of paratype males.** Similar to females except sometimes frons and vertex darker in color than remainder of head; antennae with 26 flagellomeres tapering apically; pronotum foveate to smooth at propleural margin; mesoscutum sometimes mottled orange/brown/black with or without lighter orange patches at anterior notauli; median area of mesoscutum without well-defined parallel grooves appearing more areolate-rugose; HW 0.7–0.8 mm; HL 0.6–0.68 mm; HW/HL 1.16–1.2; BL 2.5–2.86 mm; FWL 2.0–2.13 mm; CL 1.2–1.36 mm; CW 0.4 mm; CL/CW 3.3–3.4.

#### Material examined.

Holotype female: PUNTARENAS, R.F. Golfo Dulce, 3 km SW Rincon, 10 m, xii.1989–iii.1990 (P. Hanson) [UWIM]. Paratype females: 1♀, GUANACASTE, Est. Los Almendros, 300 m, Amarilla, 6–29.i.1995 (E.E. Lopez) LN 334850, 369500 #4785 [INBio]; remainder of paratype females are from PUNTARENAS province; 1♀, P. N. Corcovado, Est. Sirena, 50 m, iv–viii.1989; 2♀, same data as holotype; 1♀, same data as holotype except vi–viii.1989; 1♂, same data as holotype except S Rincon, iii–v.1989; 1♀, same data as holotype except iii–v.1989; 1♀, same data as holotype except x–xii.1990; 2♀, same data as holotype except vi.1991; 2♀, same data as holotype except i.1992; 1♀, same data as holotype except iii.1993; 4♀, same data as holotype except iv.1993; 8♀, same data as holotype except primary forest, xii.1992; 11♀, same data except ii.1993; 1♀, Puerto Jimenez, 10 m, full sun grassy weedy site, x.1991; 1♀, 1♂, same data except grassy weedy site, i–ii.1992; 1♀, 23 km N Puerto Jimenez, La Palma ,10 m, in large trees, viii–ix.1991; 2♀, 5 km N Puerto Jimenez 10 m, iii–iv.1991; 2♂, Pen. Osa, Puerto Jimenez, 10 m, ii–iii.1993; 2♀, Pen. Osa, Cerro Rincon, 200 m, S del Hito, 745 m, i.1991 (Hanson & Quiros); 1♀, same data except ii.1991 (Hanson & Godoy); 2♀, San Vito Est. Biol. Las Alturas, 1500 m, xii.1991.

#### Remarks.

*Leptodrepana sorayae* may be easily distinguished from other Costa Rican species because of its many unique characters. The configuration of the carapace apex is diagnostic for this species. In lateral view, the carapace bears a rounded flange posterior to the dorso-apical point (Fig. 105). In posterior view the carapace apex terminates in a single broad point below which is a strongly arched medially notched flange. The carapace is areolate-rugose from base to apex. The wings also have a characteristic pattern. They are suffused with smoky brown pigmentation except for a white band running from the and including the parastigma over veins RS+Ma, RS+Mb, 1m-cu, 2Cua and basal most portions of veins 2RS, 2M and 3CU (Fig. 128).

#### Etymology.

This species name is a patronym named in honor of a sister of SDD, Soraya Elizabeth Dadelahi.

### Leptodrepana soussanae

Taxon classificationAnimaliaHymenopteraBraconidae

Dadelahi & Shaw sp. n.

http://zoobank.org/C6354D5D-5139-4A20-8A59-DE1416EACBE5

Figs 107–111

#### Diagnosis.

Body robust, over 3mm in length. In posterior view apex of carapace arched; in dorsal and lateral views, apex rounded. Flagellum with uniform thickness and densely setose. Wings obfuscate except for circular area below the stigma suffused with brown pigmentation. Body mostly black, except basal fourth of carapace yellowish white.

**Figures 107–111.**
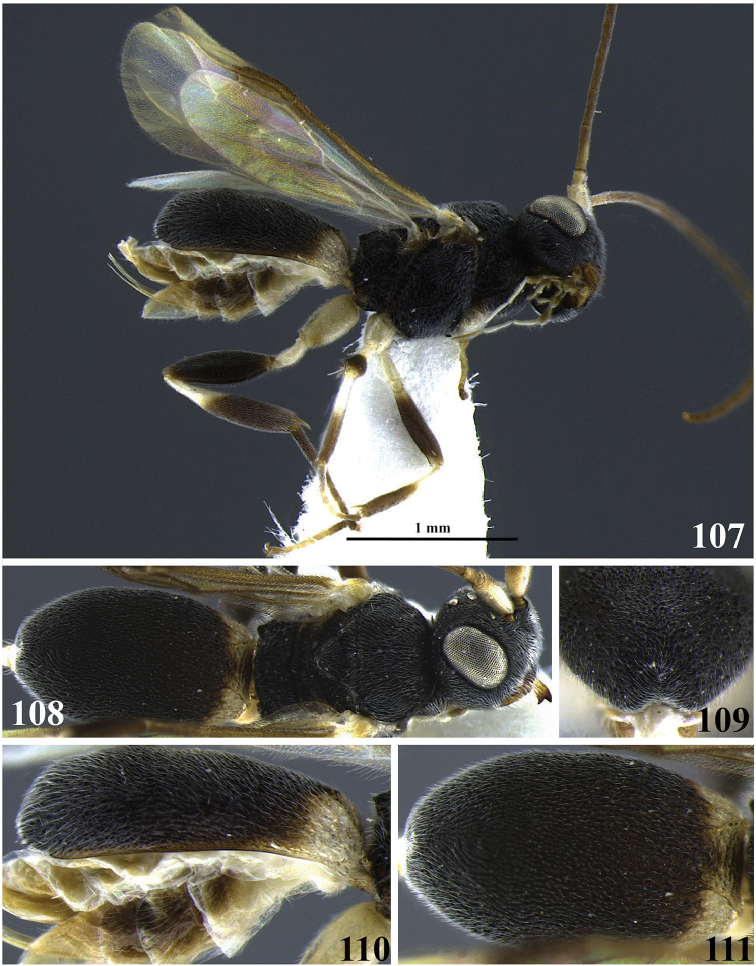
*Leptodrepana soussanae*. **107** Female habitus in lateral view **108** female habitus in dorsal view **109** metasoma in dorso-posterior view with a couple of terminal projections **110** metasoma in lateral view **111** metasoma in dorsal view.

#### Holotype female.

BL 2.93 mm; FWL 2.4 mm; CL 1.28 mm; CW 0.4 mm; CL/CW 3.2.

#### Description.

*Color*. Head black, clypeus orangish brown with yellow at apical margin; mandibles mostly yellowish brown but blackish brown basally and apically; palpi yellowish white; antennae light brown with scape, pedicel and first 2 flagellomeres (basal) yellowish white; mesosoma black; fore leg with coxa, trochanter, and trochantellus white-yellow, femur brown, tibia and tarsus white-yellow, tarsal claw brown; middle and hind leg same as foreleg except tibia brown with basal band of yellowish white and tarsus brown; wings obfuscate except for circular area below stigma suffused with brown pigmentation and densely setose covering apical half of 1^st^ submarginal cell and most of 2^nd^ submarginal cell; carapace mostly black, basal fourth of carapace yellowish white.

*Head*. HW 0.9 mm; HL 0.68 mm; HW/HL 1.33; head densely pubescent; face, genae, vertex and ocellar triangle rugulose punctate; frons depressed and median carina largely obscured by rugulose sculpture; clypeus punctate and apical margin rounded; occipital carina complete.

Mesosoma. Pronotum foveate; propleuron weakly areolate-rugose; mesoscutum densely pubescent and medially with irregular pitted grooves appearing areolate-rugose; notauli narrow and visible anteriorly; mesoscutal lobes rugulose-punctate; scutellar sulcus with five well-defined depressions, all longer than wide; scutellar disc pubescent and apparently granulate-punctate; mesopleuron anteriorly rugose, medially foveate, with wide deeply foveate groove at precoxal sulcus and foveolate-punctate below; mesopleural scrobiculate groove proximal metapleuron wide; propodeum coarsely areolate-rugose with distinct transverse carina raised into small and roughly equal medial and lateral flanges.

*Metasoma*. Carapace areolate-rugose to weakly areolate-rugose at apex; in posterior view apex arched; in dorsal and lateral views, apex rounded.

#### Variation.

**Paratype females.** HW 0.9 mm; HL 0.68–0.7 mm; HW/HL 1.28–1.33; BL 2.93–3.06 mm; FWL 2.4 mm; CL 1.28–1.4 mm; CW 0.4–0.44 mm; CL/CW 3.18–3.2.

**Paratype males.** No males.

#### Material examined.

Holotype female: GUANACASTE, Santa Rosa National Park, Bosque San Emilio 30-year-old, in clearing fully insolated part of the day, 300 m, 1.vi–22.vi.1985 (I. Gauld & D. Janzen) [UWIM]. Paratype female: 1♀, same data except old forest more or less fully shaded as possible, 14.vi–5.vii.1986.

#### Remarks.

*Leptodrepana soussanae* is superficially similar to *L. thema* but may be distinguished from this species by its robust body, over 3mm in length, uniform thickness of the flagellum and the densely setose circular area below the stigma suffused with brown pigmentation and covering apical half of 1^st^ submarginal cell and most of 2^nd^ submarginal cell. *Leptodrepana thema* is small, less than 2.5 mm. The flagellum appears slightly dilated medially. The wings are lightly suffused with yellowish brown pigment and do not have a densely setose darkly pigmented patch below the stigma.

#### Etymology.

This species name is a patronym in honor of a sister of SDD, Soussan Parker Dadelahi.

### Leptodrepana stasia

Taxon classificationAnimaliaHymenopteraBraconidae

Dadelahi & Shaw sp. n.

http://zoobank.org/599BA93F-0F9C-4AD1-857D-73A1BD08382D

Figs 112–115

#### Diagnosis.

Presence of 2 tubercles on the apex of the carapace. The penultimate flagellomere is almost the same length as the ultimate flagellomere. The carapace is completely brownish black; head and carapace brownish black; mesosoma mostly orange, anteriorly and ventrally blackish; wings lightly pigmented with yellowish brown venation.

**Figures 112–115.**
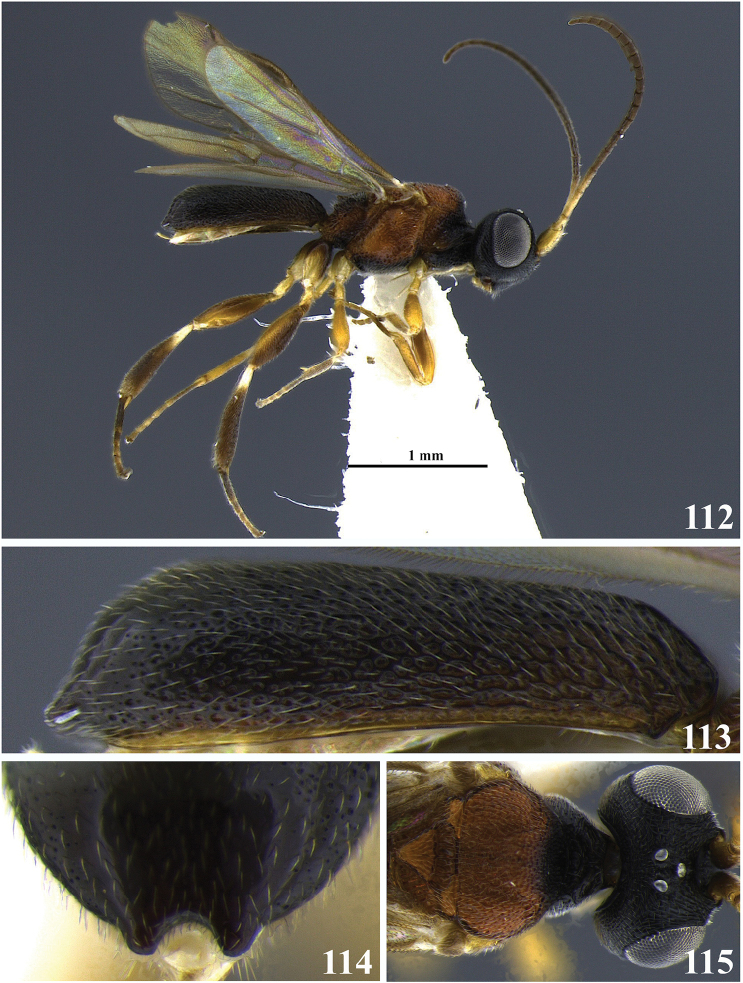
*Leptodrepana stasia*. **112** Female habitus in lateral view **113** metasoma in lateral view **114** metasoma in dorso-posterior view with a couple of pointing-terminal projections **115** head and mesoscutum in dorsal view.

#### Holotype female.

BL 2.53 mm; FWL 2.13 mm; CL 1.16 mm; CW 0.36 mm; CL/CW 3.2.

#### Description.

*Color*. Head brownish black, mandibles yellow, blackish brown apically; palpi yellowish white; antennae brown with scape, pedicel and first flagellomere yellowish white; mesosoma mostly orange, median mesonotal lobe black, postero-ventral portion of mesopleuron black, posterior metapleuron black; propodeum orangish black; middle and fore leg coxae yellowish white, remainder of legs brown; hind leg coxa brown basally and yellowish brown apically, trochanter yellowish brown, femur and tibia brown and tibia with basal white band; wings lightly pigmented with yellowish brown venation; carapace blackish brown.

*Head*. HW 0.73 mm; HL 0.6 mm; HW/HL 1.22; face, genae, vertex and ocellar triangle rugulose-punctate; frons depressed, rugulose-punctate and median carina present; clypeus punctate and apical margin rounded; occipital carina complete.

*Mesosoma*. Pronotum foveate antero-laterally to rugose-punctate at propleural margin; propleuron weakly areolate-rugose; mesoscutum medially with irregular parallel pitted grooves between notauli difficult to distinguish and appears areolate-rugose; notauli visible anteriorly; median and lateral mesonotal lobes rugose-punctate; scutellar sulcus with 6 well-defined depressions, all longer than wide; scutellar disc punctate; mesopleuron anteriorly rugose foveolate, medially weakly punctate, narrow foveate band at precoxal sulcus, foveolate postero-ventrally; propodeum coarsely areolate-rugose with distinct transverse carina raised into small and roughly equal medial and lateral flanges.

*Metasoma*. Carapace areolate-rugose to punctate at apex; in posterior and dorsal views, apex terminating in two thick tubercles close together; in lateral view, carapace terminates in narrow sloping point below midline.

**Variation of paratype females.** Metapleuron orange and black; lateral pronotum orange and black; HW 0.73–0.75 mm; HL 0.63 mm; HW/HL 1.16–1.2; BL 2.5–2.8 mm; FWL 2.13–2.3 mm; CL 1.16 mm; CW 0.32–0.36 mm; CL/CW 3.2–3.63.

**Paratype males.** No males.

#### Material examined.

Holotype female: GUANACASTE, Arenales W side Volcan Cacao, 900 m, 1988–1989 (P. Hanson) [UWIM]. Paratype data: 1♀, same data as holotype.

#### Remarks.

*Leptodrepana stasia* is superficially similar to both *L. ninae* and *L. conleyae* in color and the presence of 2 tubercles on the apex of the carapace. However it may be easily distinguished from both of these species. In *L. stasia* the penultimate flagellomere is almost the same length as the ultimate flagellomere. The carapace is completely brownish black. In both *L. ninae* and *L. conleyae* the penultimate flagellomere is almost the same length as width and approximately half the length of the ultimate flagellomere. Additionally, the carapace of both species is yellowish white basally.

#### Etymology.

This species name is an arbitrary arrangement of letters to form a euphonious combination.

### Leptodrepana strategeri

Taxon classificationAnimaliaHymenopteraBraconidae

Dadelahi & Shaw sp. n.

http://zoobank.org/111C5EB3-3ED3-4AA0-A48E-B07ED859A770

Figs 116–120

#### Diagnosis.

In dorsal and posterior views, apex rounded; in lateral view, apex terminates in broad rounded point. The scutellar sulcus has 4–5 well-defined depressions and the mesopleuron has a scrobiculate groove at the precoxal sulcus. In females, the flagellum is light brown. Body mostly blackish brown except carapace with basal third yellowish white.

**Figures 116–120.**
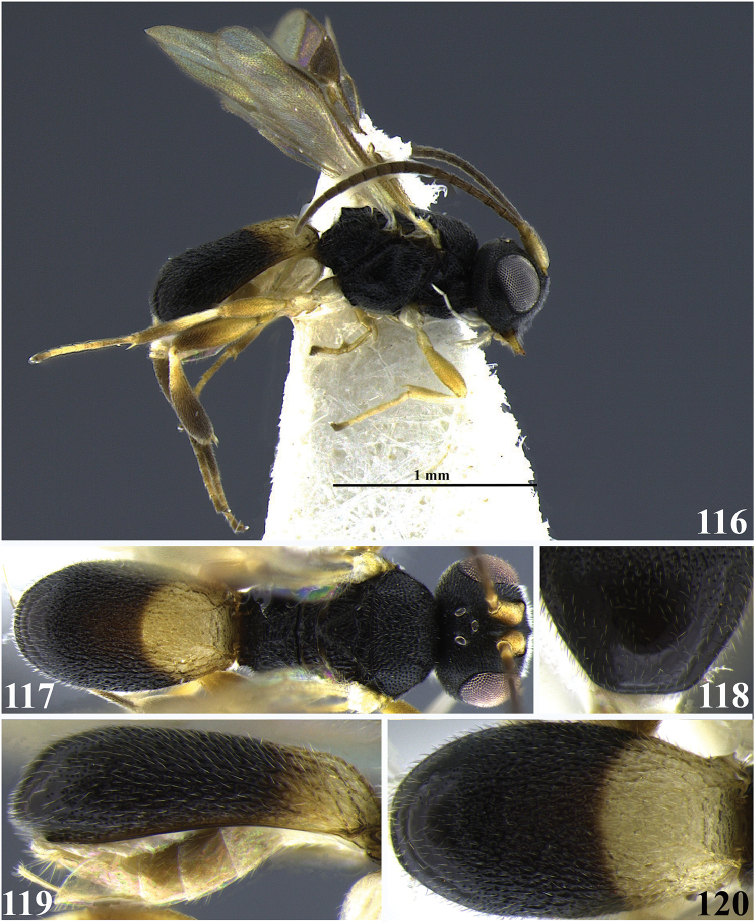
*Leptodrepana strategeri*. **116** Female habitus in lateral view **117** female habitus in dorsal view **118** metasoma in dorso-posterior view terminating truncated **119** metasoma in lateral view **120** metasoma in dorsal view.

#### Holotype female.

BL 2.13 mm; FWL 1.73 mm; CL 0.92 mm; CW 0.28 mm; CL/CW 3.28.

#### Description.

*Color*. Head black, clypeus brown, and mandibles yellow, brown apically; palpi yellowish white; antennae light brown with scape and pedicel yellowish white; mesosoma black; legs mostly yellowish brown except coxa, trochanter, trochantellus and basal portions of femur and tibia yellowish white; wings lightly pigmented with darker area below stigma covering apical half of 1^st^ submarginal cell and anterior portion of 2^nd^ submarginal cell; yellowish brown venation; carapace blackish brown with basal third yellowish white.

*Head*. HW 0.63 mm; HL 0.45 mm; HW/HL 1.39; face finely punctate, genae, vertex and ocellar triangle rugulose-punctate; frons depressed, impunctate and median carina lacking; clypeus finely punctate and apical margin rounded; occipital carina complete; antennae with flagellum slightly dilated medially.

*Mesosoma*. Pronotum densely foveolate antero-laterally to punctate at propleural margin; propleuron foveolate to weakly areolate-rugose; mesoscutum medially with irregular pitted grooves between notauli so that area appears areolate-rugose; notauli narrow and visible anteriorly; median and lateral mesonotal lobes densely foveolate; scutellar sulcus with 4–5 well-defined depressions, length almost equal to width; scutellar disc foveolate; mesopleuron anteriorly rugose-foveolate, medially shiny and weakly punctate, scrobiculate groove at precoxal sulcus and weakly punctate postero-ventrally; propodeum coarsely areolate-rugose with distinct transverse carina raised into small and roughly equal medial and lateral flanges.

*Metasoma*. Carapace areolate-rugose to shiny and weakly punctate at apex; in dorsal and posterior views, apex rounded; in lateral view, apex terminates in broad rounded point.

**Variation of paratype females.** Color: frons with faint median carina more visible anteriorly than posteriorly; first basal flagellomere yellowish brown; HW 0.63–0.65 mm; HL 0.5–0.53 mm; HW/HL 1.19–1.3; BL 2.13–2.2 mm; FWL 1.73–1.87 mm; CL 0.92–0.96 mm; CW 0.28–0.32 mm; CL/CW 2.88–3.43.

**Paratype males.** No males.

#### Material examined.

Holotype female: SAN JOSE, Ciudad Colon, 80 m, xii.1989–iii.1990 (L. Fournier) [UWIM]. Paratype data: 1♀, GUANACASTE, Arenales, W. side of Volcan cacao, 900 m, xi–xii.1990; 1♀, same as holotype; 2♀, same data except iii–iv. 1990; 2♀, same data except iv–v.1990.

#### Remarks.

Superficially *L. strategeri* is similar to *L. ronnae* but may be distinguished from this species by the lack of a transverse carina at the carapace apex in posterior view. The carapace is shiny and weakly punctate at the apex. The scutellar sulcus has 4–5 well-defined depressions and the mesopleuron has a scrobiculate groove at the precoxal sulcus. In females, the flagellum is light brown. *Leptodrepana ronnae* has a transverse carina at the carapace apex in posterior view and the apex appears rugulose. The scutellar sulcus has only three well-defined depressions and the mesopleuron has shallow irregularly shaped pits at the precoxal sulcus. In females the flagellum is brown but bears a yellowish white annulus at flagellomeres 4–6.

#### Etymology.

This species name is an arbitrary arrangement of letters to form a euphonious combination.

### Leptodrepana thema

Taxon classificationAnimaliaHymenopteraBraconidae

Dadelahi & Shaw sp. n.

http://zoobank.org/FDB0797C-B7A9-424D-B5F6-FE81FC47030E

Figs 121–125

#### Diagnosis.

Small body size, less than 2.5 mm. In posterior, dorsal, and lateral views, carapace apex rounded. The flagellum appears slightly dilated medially. The wings are lightly suffused with yellowish brown pigment and do not have a densely setose darkly pigmented patch below the stigma.

**Figures 121–125.**
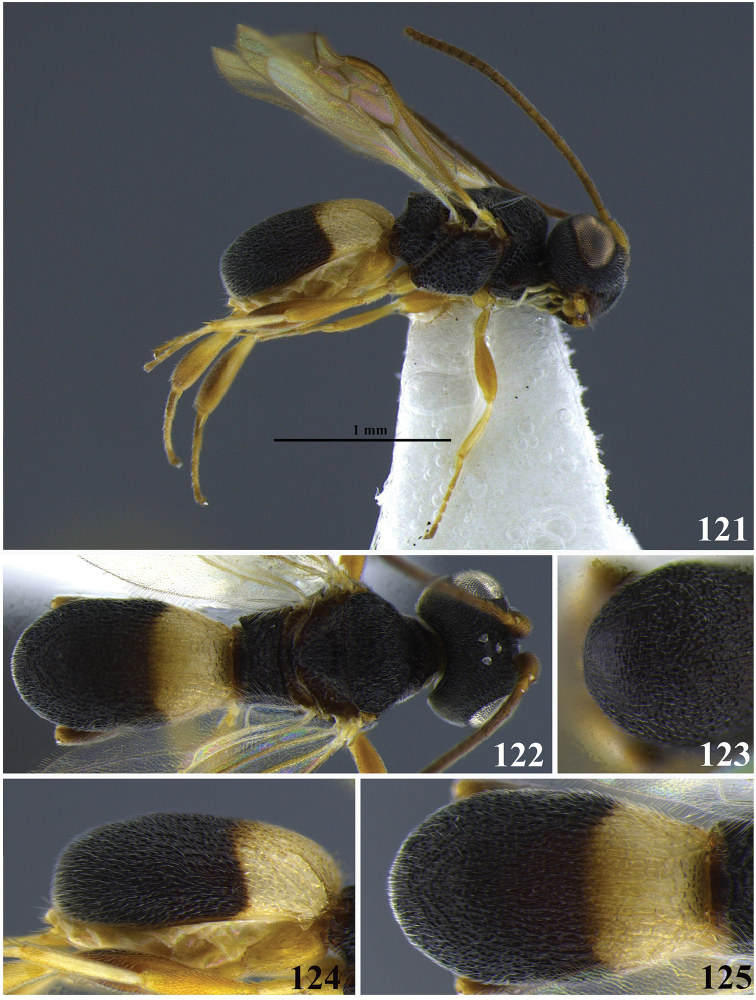
*Leptodrepana thema*. **121** Female habitus in lateral view **122** female habitus in dorsal view **123** metasoma in dorso-posterior view terminating rounded **124** metasoma in lateral view **125** metasoma in dorsal view.

**Figures 126–128.**
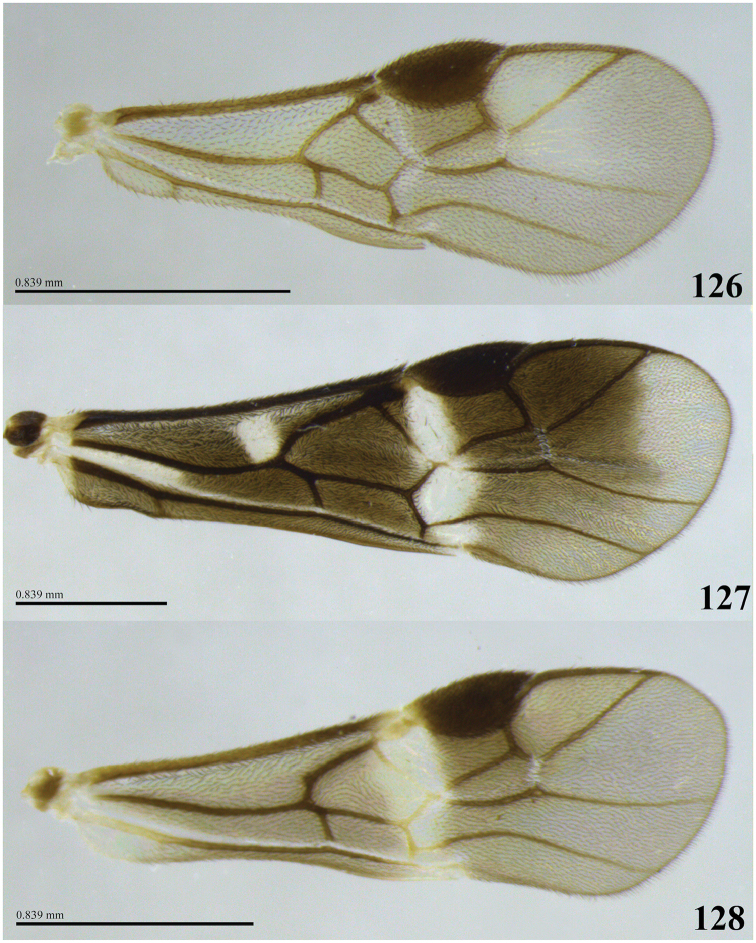
Wing color patterns in *Leptodrepana*. **126** *Leptodrepana alexisae*. **127** *Leptodrepana rosanadana*. **128** *Leptodrepana sorayae*.

#### Holotype female.

BL 2.06 mm; FWL 1.87 mm; CL 0.92 mm; CW 0.36 mm; CL/CW 2.56.

#### Description.

*Color*. Head blackish brown, mandibles yellow, blackish brown apically; apical margin of clypeus yellowishish brown; palpi yellowish white; antennae with scape and pedicel a yellowish brown and flagellum brown; mesosoma blackish brown; middle and fore legs yellow; hind leg mostly yellow except apical portions of femur and tibia yellowish brown; wings suffused with light yellowish brown pigment; venation yellowish brown; basal third of carapace yellowish white and apical 2/3 of carapace blackish brown.

*Head*. HW 0.68 mm; HL 0.53 mm; HW/HL 1.58; face, genae, vertex and ocellar triangle coarsely rugulose-punctate; frons depressed, coarsely rugulose-punctate with fine parallel lineation lateral to median carina; clypeus punctate and apical margin rounded; occipital carina complete; antennae with a slightly dilated appearance medially.

*Mesosoma*. Pronotum deeply foveate antero-laterally to weakly areolate-rugose at propleural margin; propleuron weakly areolate-rugose; mesoscutum areolate-rugose, mesonotal lobes not differentiated from median mesoscutum; notauli indistinct and visible anteriorly; scutellar sulcus with five well-defined depressions, all longer than wide; scutellar disc rugose-punctate; mesopleuron anteriorly rugose, medially coarsely foveate, foveate groove at precoxal sulcus, foveate postero-ventrally; propodeum coarsely areolate-rugose with distinct transverse carina raised into small and roughly equal medial and lateral flanges.

*Metasoma*. Carapace completely areolate-rugose; in posterior, dorsal, and lateral views, carapace apex rounded.

**Variation of paratype females.** HW 0.7–0.75 mm; HL 0.575 mm; HW/HL 1.21–1.30; BL 2.26–2.4 mm; FWL 1.86–2.06 mm; CL 0.96–1.04 mm; CW 0.4–0.44 mm; CL/CW 2.36–2.4.

**Paratype males.** No males.

#### Material examined.

Holotype female: GUANACASTE, P. N. Santa Rosa, Bosque San Emilio, 50 year old deciduous forest in clearing fully isolated part of day, 300 m, 24.v–14.vi.1986 (I. Gauld & D. Janzen) [UWIM]. Paratype data: 1♀, same data as holotype; 1♀, same data except 31.i–21.ii.1987; 1♀, same data except site #2, open regenerating woodland, 10 years old, more or less fully shaded as possible, 6–27.ix.1986.

#### Remarks.

*Leptodrepana thema* is superficially similar to *L. soussanae* but may be distinguished from this species by its small body size, less than 2.5 mm. The flagellum appears slightly dilated medially. The wings are lightly suffused with yellowish brown pigment and do not have a densely setose darkly pigmented patch below the stigma. *Leptodrepana soussanae* has a robust body, more than 3 mm in length. The thickness of the flagellum is uniform. The wing bears a densely setose circular area below the stigma that is suffused with brown pigmentation and covers the apical half of 1^st^ submarginal cell and most of 2^nd^ submarginal cell.

#### Etymology.

This species name is an arbitrary arrangement of letters to form a euphonious combination.

## Discussion

The flagellum of all female *Leptodrepana* described here is reduced to 17 flagellomeres. This reduction is also found in two N. American species described by Shaw (1983): *L. opuntiae* and *L. oriens*. Since this character is found in all female Costa Rican *Leptodrepana* it may be a synapomorphic character closely relating these species to *L. opuntiae* and *L. oriens*.

The species described in this work differ slightly from Shaw’s (1983) generic diagnosis. All females of Costa Rican *Leptodrepana* have 17 flagellomeres. The depressions of the scutellar sulcus vary from 3–8, and the propodeal tubercles are often distinct. In the original diagnosis, Shaw (1983) stated that the flagellum is sometimes reduced to 17 flagellomeres and that the scutellar sulcus usually consists of 7–10 depressions. Additionally, the diagnosis holds that the propodeal tubercles of *Leptodrepana* are usually short or indistinct.

None of the newly described Costa Rican species share the intermediate character states sometimes found in the Palearctic species. Intermediate forms are problematic but the solution is not synonymy. As noted in the introduction, inclusion of *Leptodrepana* in *Ascogaster* renders the genus paraphyletic. This is no less problematic than the question of what to do with intermediate forms between the two genera. Phylogenetic data support genus status in *Leptodrepana*, in addition to strong morphological and biological evidence (Chen 1999, Shaw 1983, 1997).

## Supplementary Material

XML Treatment for Leptodrepana

XML Treatment for Leptodrepana alexisae

XML Treatment for Leptodrepana atalanta

XML Treatment for Leptodrepana conda

XML Treatment for Leptodrepana conleyae

XML Treatment for Leptodrepana demeter

XML Treatment for Leptodrepana eckerti

XML Treatment for Leptodrepana gauldilox

XML Treatment for Leptodrepana hansoni

XML Treatment for Leptodrepana kimbrellae

XML Treatment for Leptodrepana lorenae

XML Treatment for Leptodrepana munjuanae

XML Treatment for Leptodrepana ninae

XML Treatment for Leptodrepana pamelabbas

XML Treatment for Leptodrepana ronnae

XML Treatment for Leptodrepana rosanadana

XML Treatment for Leptodrepana schuttei

XML Treatment for Leptodrepana scottshawi

XML Treatment for Leptodrepana shriekae

XML Treatment for Leptodrepana sohailae

XML Treatment for Leptodrepana sorayae

XML Treatment for Leptodrepana soussanae

XML Treatment for Leptodrepana stasia

XML Treatment for Leptodrepana strategeri

XML Treatment for Leptodrepana thema
